# Targeted enhancement of bacteriophage activity against antibiotic-resistant *Staphylococcus aureus* biofilms through an evolutionary assay

**DOI:** 10.3389/fmicb.2024.1372325

**Published:** 2024-07-08

**Authors:** Luis Ponce Benavente, Jeroen Wagemans, Dennis Hinkel, Alba Aguerri Lajusticia, Rob Lavigne, Andrej Trampuz, Mercedes Gonzalez Moreno

**Affiliations:** ^1^Corporate Member of Freie Universität Berlin and Humboldt, Center for Musculoskeletal Surgery, Charité - Universitätsmedizin Berlin, Berlin, Germany; ^2^BIH Center for Regenerative Therapies (BCRT), Berlin Institute of Health at Charité - Universitätsmedizin Berlin, Berlin, Germany; ^3^Department of Biosystems, KU Leuven, Leuven, Belgium; ^4^Institut für Chemie und Biochemie, Freie Universität Berlin, Berlin, Germany

**Keywords:** antimicrobial resistance, biofilm, bacteriophage, directed evolution, *Staphylococcus aureus*

## Abstract

*Staphylococcus aureus*´ biofilm-forming ability and rapid resistance development pose a significant challenge to successful treatment, particularly in postoperative complications, emphasizing the need for enhanced therapeutic strategies. Bacteriophage (phage) therapy has reemerged as a promising and safe option to combat multidrug-resistant bacteria. However, questions regarding the efficacy of phages against biofilms and the development of phage resistance require further evaluation. Expanding on the adaptable and evolutionary characteristics of phages, we introduce an evolutionary approach to enhance the activity of *S. aureus* phages against biofilms. Unlike other *in vitro* directed evolution methods performed in planktonic cultures, we employed pre-stablished biofilms to do a serial-passage assay to evolve phages monitored by real-time isothermal microcalorimetry (IMC). The evolved phages demonstrated an expanded host range, with the CUB_MRSA-COL_R9 phage infecting 83% of strains in the collection (*n* = 72), surpassing the ISP phage, which represented the widest host range (44%) among the ancestral phages. In terms of antimicrobial efficacy, IMC data revealed superior suppression of bacterial growth by the evolved phages compared to the ancestral CUB-M and/or ISP phages against the respective bacterial strain. The phage cocktail exhibited higher efficacy, achieving over 90% suppression relative to the growth control even after 72 h of monitoring. Biofilm cell-counts, determined by RT-qPCR, confirmed the enhanced antibiofilm performance of evolved phages with no biofilm regrowth up to 48 h in treated MRSA15 and MRSA-COL strains. Overall, our results underscore the potential of biofilm-adapted phage cocktails to improve clinical outcomes in biofilm-associated infections, minimizing the emergence of resistance and lowering the risk of infection relapse. However, further investigation is necessary to evaluate the translatability of our results from *in vitro* to *in vivo* models, especially in the context of combination therapy with the current standard of care treatment.

## 1 Introduction

The great intricacy of bacterial communities, known as biofilms, has been evolving during their existence on this planet. Bacteria within mature polymeric matrixes are protected against harsh ecological circumstances and antimicrobials (Dzianach et al., 2019). The challenge of biofilm control has an inevitable impact on our environment, food industry, water treatment and human health. *Staphylococcus aureus* is a prominent pathogen in post-surgery complications, severe infections such as bacteremia or endocarditis, and difficult-to-treat bone and joint infections (Sultan et al., 2022). Adding its ability to form biofilms and generate chronic infections in the body (Zimmerli and Sendi, 2017), along with emergence and rapid dissemination of multidrug resistance (Riya et al., 2021), current therapeutic options are becoming obsolete and the search for new targeted treatment approaches is urgent.

Bacteriophages are natural predators of bacteria, able to co-evolve with their hosts in a wide range of natural ecosystems (Scanlan et al., 2015) and co-exist with bacterial biofilms (Visnapuu et al., 2022). Phage therapy exploits the ability of phages to infect and kill bacteria in the treatment of infectious diseases. This represents a potential alternative or adjuvant to antibiotics to fight against multidrug-resistant pathogenic bacteria (Kutateladze and Adamia, 2010), but also in the treatment of biofilm-associated infections, which are difficult to eradicate with currently available antibiotics.

Several researchers described numerous applications of phage therapy (e.g., intravenous, oral, topical) providing evidence of its high safety and efficacy in animal models and humans (El Haddad et al., 2019; Melo et al., 2020; Abedon et al., 2021; Uyttebroek et al., 2022). So far, no serious side effects in phage application have been reported (Romero-Calle et al., 2019). Moreover, many phages are specific against a host or host range, infecting a limited number of strains among a single bacterial species (Sulakvelidze et al., 2001; Weinbauer, 2004). The specificity of this phage-bacteria interaction may be considered as an advantage because phages eliminate only the targeted bacterial strain, protecting normal flora colonizers unrelated to the pathogen species.

Various strategies have been suggested to enhance the currently limited therapeutic potential of wild-type phages. Among them we have genetic engineering of *E. coli* phages to target *Yersinia* and *Klebsiella* strains (Ando et al., 2015) and deletion of an endolysin in a temperate *S. aureus* phage to increase its *in vitro* and *in vivo* bactericidal activity (Paul et al., 2011). Furthermore, Burrowes et al. (2019) and Mapes et al. (2016) describe protocols to expand the host range of *P. aeruginosa* phages. Phage training tested on planktonic staphylococcal strains has been performed and even applied as therapy against a recalcitrant *S. epidermidis* infection (Doub et al., 2021). Even though the mentioned approaches seem to have clinical potential, tests focusing on biofilm models need to be included during their development.

Earlier research carried out by our group, both *in vitro* (Tkhilaishvili et al., 2018, 2020a,b) and in patients (Tkhilaishvili et al., 2019; Exarchos et al., 2020; Mulzer et al., 2020), showed that a combination of phage and antibiotic therapy can be an effective treatment for biofilm-associated infections. Nonetheless, as with antibiotics, bacteria exposed to phages can develop resistance (Aslam et al., 2020), which can lead to treatment failure. Approaches to constrain the development of phage-resistant bacteria include the use of phage cocktails as well as phage training (Yang et al., 2020; Sáez Moreno et al., 2021).

Following our previous work with *Pseudomonas aeruginosa* phages (Kunisch et al., 2023a), in this study we aimed to improve the antimicrobial capabilities of *S. aureus* phages by an evolutionary approach. Through an *in vitro* serial-passage biofilm assay and real-time isothermal microcalorimetry (IMC) monitoring, we were able to improve the antibiofilm activity of novel phages against multi-drug resistant *S. aureus* strains and increase their host range. Following a systematic characterization of the antimicrobial properties of evolved phages, we combined a cocktail of biofilm-adapted phages and quantitatively assessed its efficacy against biofilms by real time quantitative polymerase chain reaction (RT-qPCR), achieving a greater efficacy than each antimicrobial agent alone.

## 2 Materials and methods

### 2.1 Bacteria and bacteriophage collection

#### 2.1.1 Bacteria collection

A total of 72 genome-sequenced *S. aureus* clinical and laboratory standard strains collected from different research laboratories located in Switzerland (*n* = 23), Germany (*n* = 23), Belgium (*n* = 12) and Italy (*n* = 14) were included in this study. Antibiogram analysis for each strain was performed by routine microbiology laboratory. Bacterial stocks were prepared in 20% glycerol and stored at −80°C. Bacteria were cultured in Tryptic Soy Broth (TSB) (US Biological, Eching, Germany) or Müller Hinton Broth (MHB) (Roth, Karlsruhe, Germany) for liquid cultures or in TSB with addition of 15 g/L (TSA) or 6 g/L (soft agar) agar (Sigma-Aldrich, Steinheim, Germany) for solid cultures.

#### 2.1.2 Phage isolation

Sewage and human saliva samples were collected, homogenized at 24°C and aliquoted in 50 mL tubes, after which bacteria and large waste particles were removed by centrifugation for 30 min at 3,000 x g followed by successive filtration of the supernatant through a 0.45 μm and 0.22 μm membrane. Phage enrichment was performed by co-incubating 50 mL of collected sample with 50 mL of double concentrated TSB supplemented with 5 mM MgSO_4_ and 5 mM CaCl_2_ and inoculated with 100 μL of bacterial culture. After overnight incubation at 37°C, remaining bacteria were removed from the lysed cultures by centrifugation for 10 min at 3,000 x g and 4°C. The 0.22 μm-filtered enrichment solutions were then spot tested on soft agar overlays for phage identification. Plaques appearing on the plates were purified by four consecutive single-plaque-passages to ensure phage purity. Then, each isolated phage was produced from a single plaque using either a liquid (Kunisch and Moreno, 2023) or solid (Kunisch et al., 2023b) propagation method. Ultimately, phage lysates were concentrated and purified by PEG 8000 precipitation (Kunisch et al., 2023c) and stored at 4°C until further use. Three phages (CUB-A, CUB-B, and CUB-M) were isolated and characterized as a part of this study. Additionally, the *Staphylococcus* phages, myovirus ISP (NC_047720) and podovirus 44AHJD (NC_004678), provided by research partners, were also included to be part of the *in vitro* directed evolution experiment.

#### 2.1.3 Whole genome sequencing and analysis

Bacterial genomes were isolated from a single-colony overnight culture using the DNeasy UltraClean Microbial Kit (Qiagen, Hilden, Germany) following the manufacturer’s instructions. The purity and concentration of the extracted genomes were assessed with NanoDrop 1,000 spectrophotometer (PEQLAB Biotechnologie GmbH, Erlangen, Germany). Subsequently, a DNA library was prepared with the Nextera Flex DNA Library Prep kit (Illumina, San Diego, CA, USA) and sequenced on an Illumina MiniSeq instrument with the MiniSeq High Output Reagent Kit (paired-end, 2 × 150 bp). Moreover, the Rapid Barcoding Kit (Oxford Nanopore Technology, Oxford, UK) was applied to prepare the same DNA samples for long-read sequencing on a MinION device using an R9.4.1 flowcell (Oxford Nanopore Technology). Guppy v3.1.5 was employed as basecaller. Then, the Unicycler hybrid assembly pipeline v0.4.8.0 (Wick et al., 2016) was executed to assemble the bacterial genomes. The raw sequencing reads data of all 72 strains and their genome assemblies were submitted to NCBI and are accessible under BioProject PRJNA952137. The quality of the assembly was visualized with Bandage v0.8.1 (Wick et al., 2015). Next, the genomes were functionally annotated using Prokka v1.14.6 (Seemann, 2014). The core genome was determined using Roary v3.13.0 (Page et al., 2015) and a maximum likelihood phylogenetic tree was inferred using RAxML v8.2.4 (Stamatakis, 2014) for the complete *S. aureus* collection, which was then visualized with iTOL v6.5 (Letunic and Bork, 2019).

Phage genome extraction and quality control of newly isolated phages (CUB-A, CUB-B, and CUB-M) were performed as previously described (Wang et al., 2020). The phage DNA was then sequenced with Illumina similarly as for the bacterial DNA samples. The phage genomes were assembled using the SPAdes-based PATRIC genome assembly v3.6.12 (Bankevich et al., 2012). BLASTn v2.13.0 (Zhang et al., 2000) was used to find the closest similar phages in the database. Genome alignment to these identified phages was performed using MEGA11 (Tamura et al., 2021). Resulting aligned phage genomes were functionally annotated through the RASTtk pipeline and manually curated using BLASTp v2.13.0 (Altschul et al., 1997). Genbank files were finalized using Artemis v18.1.0 (Rutherford et al., 2000) and deposited in the NCBI database under accession codes OR495699, OR495700 and OR495701. To visualize phage genomes, linear comparison figures were created using Easyfig v2.2.2 (Sullivan et al., 2011).

#### 2.1.4 Phage morphology by transmission electron microscopy

The phage morphologies were visualized by transmission electron microscopy (TEM) using negative staining. An aliquot of 15 μL of the purified phage particle preparation was dropped onto Parafilm before transferring it onto a Ni-mesh grid (G2430N; Plano GmbH, Wetzlar, Germany) which has been carbon-coated and glow discharged (Leica MED 020, Leica Microsystems, Wetzlar, Germany). The phage particles were allowed to adsorb for 10–15 min at room temperature. The grids were washed three times with Aquadest and then treated with 1% aqueous uranyl acetate (SERVA Electrophoresis GmbH, Heidelberg, Germany) for 20 s for negative staining followed by the removal of excess staining with filter paper. Grids were air dried and then imaged by TEM using a Zeiss EM 906 microscope (Carl Zeiss Microscopy Deutschland GmbH, Oberkochen, Germany) at a voltage of 80 kV.

### 2.2 *In vitro* bacteriophage experimental evolution

#### 2.2.1 Biofilm formation and imaging

Sterile porous glass beads (Ø 4 mm, ROBU, Hattert, Germany) were used as surface for biofilm formation. Beads were incubated in 24-well-plates (one bead per well) containing 1 mL TSB inoculated with a 1:100 dilution of a single-use glycerol stock of the corresponding *S. aureus* strain at 37°C and 150 rpm orbital shaking in humid conditions for 24 h. Sterility controls containing beads in 1 mL sterile media were included.

Scanning electron microscopy (SEM) was performed for visualization of biofilms formed on glass beads. Biofilm-beads were first dip-washed in phosphate buffered saline (PBS) (Merck KGaA, Darmstadt, Germany) and then fixated in a solution of 1% paraformaldehyde and 2.5% glutaraldehyde in 50 mM HEPES for 48 h at room temperature. All samples were washed afterwards in 50 mM HEPES, dehydrated in consecutive steps of 30, 50, 70, 90, 95, 100 and again 100% ethanol, chemically dried overnight in bis (trimethylsilyl) amine (Sigma-Aldrich, Darmstadt, Germany), mounted on aluminum stubs, sputter coated with a 16 nm layer of gold–palladium, and examined in the SEM (ZEISS 1530 Gemini, Carl Zeiss Microscopy GmbH, Germany) operating at 3 kV using the SE2 electron detector.

#### 2.2.2 Evolution assay setup

**Figure 1:**
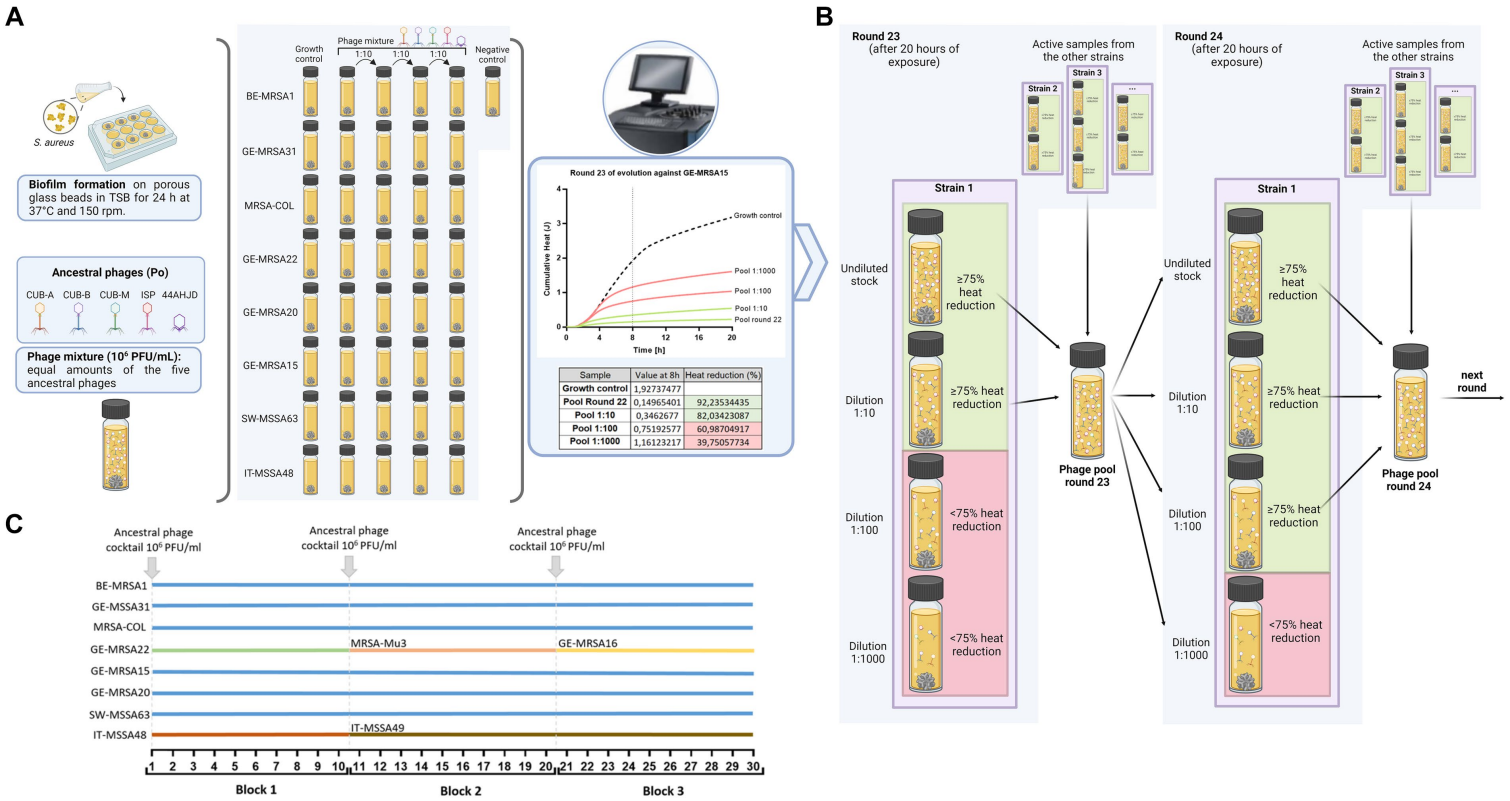
Representation of the evolution assay setup and the selection of active samples. **(A)** Biofilm formation using porous glass beads and creation of phage mixture containing equal amounts of the five ancestral phages for round 0. A pre-formed biofilm bead of each strain was placed into individual glass ampules containing the phage mixture at different titers and incubated in the IMC at 37°C for 20 h. Calorimetry data was collected in real time and heat reductions were calculated for each sample at the end of the monitored time, respective to the corresponding growth control (GC) sample. As an example, heat reductions from round 23 for strain GE-MRSA15 is displayed on the plotted graph. **(B)** Representation of active sample selection after collecting data. A percentage of heat reduction ≥75% after the initial 8 h of monitored was used to define an active sample (green), whereas samples with <75% heat reduction were considered inactive (red). All active samples from all bacterial strains exposed to phages at that round were pooled together (e.g., phage pool of round 23) and used to initiate the next round of evolution (round 24 in our example). **(C)** Scheme of included bacterial strains throughout the evolution assay consisting of three blocks, each comprising 10 consecutive rounds. At the onset of each block, new bacterial strains were exposed to the ancestral phage mixture for one round. Strains depicted with the blue line were present for all 30 rounds, whereas some strains were present during 10 (GE-MRSA22, MRSA-Mu3, GE-MRSA16 and IT-MSSA48) or 20 (IT-MSSA49) rounds. Figure was created with BioRender.com.

*In vitro* phage evolution consisted of a serial passaging approach with 30 consecutive rounds. In each 20 h round, the undiluted and three tenfold serial dilutions of a mixture of phages were independently co-incubated with 24-h-old biofilms of each *S. aureus* strain (BE-MRSA1, GE-MSSA31, MRSA-COL, GE-MRSA22, GE-MRSA15, GE-MRSA20, SW-MSSA63, IT-MSSA48, MRSA-Mu3, IT-MSSA49, GE-MRSA16) formed on glass beads. A phage mixture composed of equal amounts of the five ancestral phages (CUB-A, CUB-B, CUB-M, ISP and 44AHJD) was prepared at a final total titer of 10^6^ PFU/mL to initiate the first round (R0) of evolution. Prior to each consecutive evolution round, a new phage mixture was prepared combining all active samples from the previous round. A schematic illustration of the *in vitro* evolution experiment is depicted in Figure 1.

The inclusion criteria for the ancestral phages to be part of the evolution assay were (i) a strictly lytic lifecycle and (ii) diverse phage taxonomy, including myovirus and podovirus morphologies. Inclusion criteria for the bacterial strains were (a) susceptibility to at least one of the ancestral phages, (b) genomic diversity to represent the different lineages of the phylogenetic tree, (c) ability to form biofilm (as visualized by SEM) and (d) diverse antibiotic resistance profile.

An isothermal microcalorimeter (TAM III; TA Instruments, New Castle, DE, USA), set at a constant temperature of 37°C, was employed to monitor in real-time the heat production by each sample in each round. For each bacterial strain, the biofilm not exposed to phages was included as growth control (GC) reference. A sterility control, consisting of TSB and a sterile glass bead, was also included in every round. All samples were prepared in 4 mL airtight glass ampoules (Waters GmbH, Eschborn, Germany).

After each round, active samples were defined based in terms of a heat reduction (J) of ≥75% in comparison to the equivalent growth control sample at the initial eight-hour monitoring. The threshold of 75% was set as a conservative value, high enough to reveal a noticeable reduction in activity but sufficiently low to avoid excessive exclusion of samples, which could result in an overly high loss of phage diversity, especially in the early rounds of evolution. All active samples and samples containing the undiluted phage mixture from all bacterial strains were pooled together in a single mixture after each round. The pooled solution was centrifuged at 7,000 rpm (6,026 g) for 20 min and the supernatant filtered (0.22 μm) before being applied to the next round of evolution. The phage titer of the pooled solution was determined on each bacterial strain by a spot assay prior to each consecutive round.

Throughout the evolution assay, for each bacterial strain, the following criteria were applied to define what phage-mixture-dilution should be included at the subsequent round:

1. The undiluted phage sample was always included for each round.
2. If two consecutive dilutions reached ≥75% heat reduction, a further dilution was included for the following round.
3. if samples showed <25% heat reduction or heat reductions comparable to those observed on round 1, in 10 consecutive rounds of evolution, the respective host bacterial strain was exchanged to encourage new phage-bacteria interactions that could lead to further antimicrobial enhancement of the phages.
4. If criteria 2 or 3 did not apply, then the tenfold dilutions did not vary for the next round and the strain was kept during all 30 rounds of evolution.

Any new bacterial strain incorporated into the evolution assay was exposed for one round to the ancestral phage mixture (t_0_).

### 2.3 Re-isolation and characterization of evolved bacteriophages

Upon completion of 10, 20 and 30 rounds of evolution, and aiming to retrieve the phages with the highest antimicrobial activity, evolved phages were re-isolated from the pooled-phage-mixture that corresponded to the evolution round showing the highest heat reduction within the evolution block. Then, serial dilutions of the respective phage mixture were spotted on soft agar overlays from four bacterial strains present in the 30 rounds of evolution (BE-MRSA1, GE-MRSA15, GE-MSSA31 and MRSA-COL). Individual phages were re-isolated from each plate, randomly picking different plaques, purified, and produced as previously described. A total of 15 phages isolated from block 1 (*n* = 5), block 2 (*n* = 4) and block 3 (*n* = 6) were further characterized. Evolved phages were named after the bacterial strain of isolation (e.g., GE-MRSA15) and the round of evolution it was isolated from (e.g., “R7” for round 7).

Whole genome sequencing of each evolved phage was performed as described above and compared to the genome sequence of ancestral phages using BLASTn to determine descendancy lines. Snippy tool was then used to accurately identify and analyze single nucleotide polymorphisms (SNPs) within the genomic data.

### 2.4 Antimicrobial activity assessment

#### 2.4.1 Host range

The host range of the five ancestral phages and the 15 evolved phages was evaluated against the collection of 72 *S. aureus* strains by soft agar overlay spot assays. To this end, 400 μL of an overnight bacterial culture were mixed with 10 mL of molten soft agar (54°C) and poured over a TSA square plate to produce a homogeneous bacterial lawn. Phages were tenfold diluted (10^−3^ – 10^−9^) in 96-well microplates and 5 μL of each dilution was spotted on the overlay. Plates were let to dry for 10 min and then incubated at 37°C overnight. Bacterial strains were considered susceptible to a phage when single phage plaques were visible within any of the dilutions, but not if only a lysis zone without individual plaques was observed, to avoid false positive results due to lysis from without (Mapes et al., 2016). The experiment was carried out in biological triplicates for the ancestral phages and in two biological replicates using technical duplicates with the evolved phages. Technical replicates were performed using independent serial dilutions.

#### 2.4.2 Isothermal microcalorimetry and phage analysis

IMC was used for a comparative analysis of the antimicrobial activity against biofilm between evolved phages isolated in different evolutionary blocks and the ancestor phage. To that end, two representative *S. aureus* strains, GE-MRSA15 and MRSA-COL (both included in the evolution), were used for comparative testing of the antimicrobial activity of the evolved phages isolated using these strains (CUB_GE-MRSA15_R7/R14/R23 and CUB_MRSA-COL_R9/R20/R23) and the ancestral phages. In addition, the antimicrobial effect of a phage cocktail comprising two evolved phages (CUB_GE-MRSA15-R14 and CUB_MRSA-COL_R23) showing high efficacy was also investigated against the two bacterial strains.

First, biofilm-embedded glass beads were formed for each bacterial strain as described above and added into 4 mL glass ampoules containing 1 mL sterile TSB inoculated with the respective phage (10^7^ PFU/mL), or without phage (GC), and heat production was monitored during 72 h. The experiments were performed as two biological replicates with two technical replicates each. Antimicrobial efficacy was evaluated in terms of percentage of heat reduction of phage-containing samples relative to GC samples determined at 24 h, 48 h and 72 h time point. The statistical analysis was performed with GraphPad Prism 8.0 (GraphPad Software, La Jolla, CA, USA). A t-test with Welch correction was employed.

#### 2.4.3 Real-time quantitative PCR evaluation

Following the evaluation of the antimicrobial efficacy of the evolved phages by IMC, the phage cocktail composed of a 1:1 ratio of two evolved phages with enhanced antimicrobial activity (CUB_GE-MRSA15_R14 and CUB_MRSA-COL_R23) was evaluated by RT-qPCR to quantitatively determine antibiofilm activity. Biofilms were exposed to each single evolved phage, to the phage-cocktail or to the ancestral ISP phage, after which a RT-qPCR was performed for the quantification of bacterial cell number reduction. The protocol was based on a previously described method (Liu et al., 2014). Briefly, 24-h-biofilms formed on glass beads were dip-washed in 2 mL PBS and transferred to 24-well plates containing 1 mL sterile TSB inoculated with phage (10^7^ PFU/mL) or without phage (GC). Plates were then incubated at 37°C and 150 rpm orbital shaking for 0 h, 4 h, 8 h, 24 h or 48 h. After incubation, each bead was dip-washed in 2 mL PBS and transferred to an Eppendorf tube containing 1 mL PBS and subsequently sonicated at 200 W and 40 kHz for 20 min. Then, the sonicated solution was used for DNA extraction of the dislodged biofilm-bacteria-cells, using the DNeasy UltraClean Microbial Kit (QIAGEN, Hilden, Germany). Extracted bacterial DNA was stored at −20°C until further use. The experiment was performed using three biological replicates.

The NZYTech *Staphylococcus aureus* Real-time PCR Kit was used according to the manufacturer’s instructions (NZYTech, Lisboa, Portugal). The extracted DNA was amplified and quantified in a Mastercycler RealPlex2 (Eppendorf, Hamburg, Germany). For each experiment, as part of the PCR kit, a positive control and negative control were included. The resulting copies were multiplied by 5 and the obtained CFU values were plotted. Results were statistically analyzed using student’s t-test using Welch correction with GraphPad Prism 8.0.

## 3 Results

### 3.1 Characterization of isolated phages: genomic and morphologic analysis

Three lytic phages targeting *S. aureus* were isolated from human saliva (CUB-M) and sewage (CUB-A and CUB-B). BLASTn analysis revealed similarity to Twort-like *Staphylococcus* phages. CUB-M displayed a high similarity to the lytic phage vBMS-A1M (MK584893.1) with 99% query coverage (QC) and 99.7% sequence identity (SI). The closest related phages to CUB-A and CUB-B were philPLA-RODI (KP027446.1; 89% QC, 97.28% SI) and SauM_0414 (MH107769.1; 94% QC, 96.25% SI), respectively. These genomic data confirm the myovirus morphology of all three isolated phages as observed with TEM (Supplementary Figure S1). Phage virions showed an eicosahedral head (CUB-M: 81.8 + 3.4 nm; CUB-A: 84 ± 1 nm; CUB-B: 82.4 ± 2.5 nm in diameter) and a contractile tail (CUB-M: 214 nm; CUB-A: 209 ± 3 nm; CUB-B: 233 ± 0.5 nm extended tail-length).

A comparative genomic analysis among the five ancestral phages included in the experimental evolution setup revealed higher similarity between myovirus phages, especially between ISP and CUB-M (93% QC and 99.95% SI) and no similarity with the podovirus phage 44AHJD (Table 1).

**Table 1:** BLASTn alignment of ancestral phages.

Ancestral phage	CUB-A (142,581 bp)	CUB-B (141,181 bp)	CUB-M (140,322 bp)	ISP (138,339 bp)
CUB-B	91% QC; 98.42% SI			
CUB-M	89% QC; 98.22% SI	90% QC; 98.35% SI		
ISP	89% QC; 98.25% SI	90% QC; 98.36% SI	93% QC; 99.95% SI	
44AHJD (16,784 bp)	0% QC; 0% SI	0% QC; 0% SI	0% QC; 0% SI	0% QC; 0% SI

bp, base pairs; QC, query coverage; SI, sequence identity.

The genomes were functionally annotated, and no proteins associated with a lysogenic lifecycle or host virulence could be identified, making these phages potential candidates for their use in phage therapy. The genomes were submitted to the Genbank database and are available under accession numbers OR495699, OR495700 and OR495701.

### 3.2 *In vitro* bacteriophage evolution assay

Our evolutionary approach based on serial passaging was designed following inclusion/exclusion criteria for the selection of the bacteria and phages to be included in the assay.

To select *S. aureus* strains for the evolution assay, a phylogenetic tree was constructed based on the alignment of the core genes from the 72 strains in our collection. Four main genomic clusters could be identified (Supplementary Figure S2) with cluster 1 (yellow) comprising 38.89% of the strains and clusters 2 (red), 3 (green) and 4 (purple) including 27.78, 18.06 and 15.28%, respectively. Initially, eight bacterial strains, belonging to different genomic clusters to reflect the diversity of strains within the collection, were selected for the evolution assay. Additionally, the selected strains presented different antibiotic resistance profiles (Supplementary Table S1) and ability to form biofilm (Supplementary Figure S3). Each selected bacterial strain was susceptible to at least one of the five ancestral phages (Supplementary Table S2).

**Figure 2:**
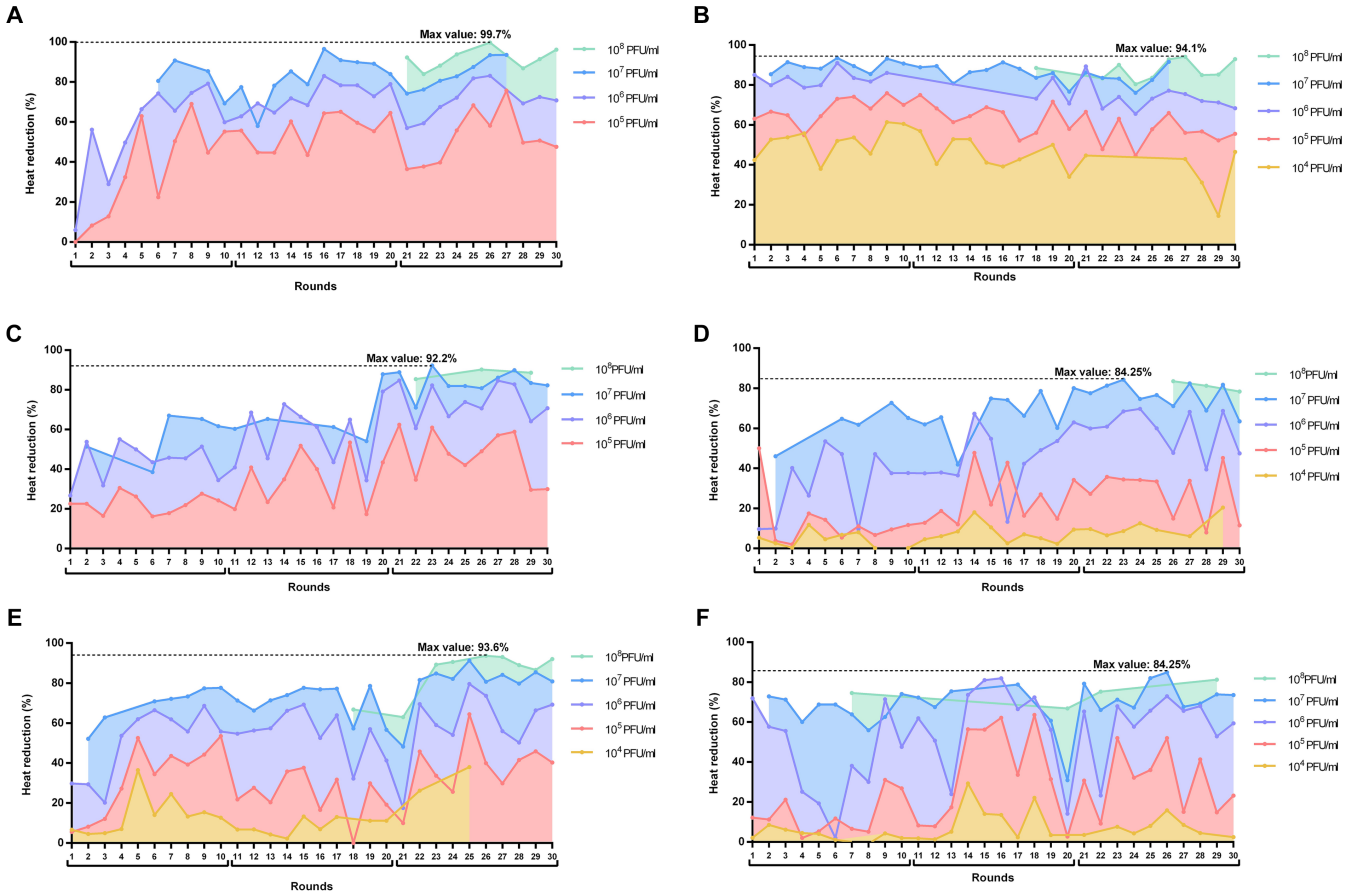
Heat reductions (%) over the 30 rounds of serial passages of BE-MRSA1 **(A)**, GE-MSSA31 **(B)**, GE-MRSA15 **(C)**, MRSA-COL **(D)**, GE-MRSA20 **(E)** and SW-MSSA63 **(F)** strains present in all rounds. The titer of the phage mixture was estimated at the onset of each round for each strain. The percentage value in each round corresponds to the heat reduction after 20 h exposure of biofilms to each phage dilution (PFU/ml) relative to the heat produced by the respective growth control (not exposed to phage). The whole experiment is divided in three blocks of 10 rounds each (block 1: rounds 1–11; block 2: rounds 11–20; block 3: rounds 21–30). Plots were prepared using GraphPad Prism 8.0.

Throughout 30 rounds of directed evolution, we observed a general trend toward an improved capability of the phage mixture to suppress the heat production of the biofilm (Figure 2), except with strain GE-MSSA31. This improvement was evidenced by generally higher percentages of heat reduction at consecutive rounds (despite a decline in heat suppression in certain rounds) and at lower phage concentrations.

When analyzing the calorimetric results after 20 h monitoring for each block (block 1: rounds 1–10; block 2: rounds 11–20; block 3: rounds 21–30), we observed that in all cases the highest heat reduction was achieved in evolution rounds belonging to the third block. The maximal heat reduction on GE-MRSA15 (Figure 2C) and MRSA-COL (Figure 2D) biofilms occurred at round 23 (92.2 and 94.25% respectively), corresponding to a 10^7^ PFU/mL titer of the phage mixture; whereas biofilm samples from BE-MRSA1 (Figure 2A), GE-MRSA20 (Figure 2E) and SW-MRSA63 (Figure 2F) exposed to 10^8^ CFU/mL of the phage mixture showed the highest heat reduction at round 26, reaching 99.7, 93.6 and 84.25% correspondingly. Finally, on GE-MSSA31 (Figure 2B) biofilm, the peak of heat reduction was reached at round 27 corresponding to a titer of 10^8^ PFU/ml of the phage mixture.

### 3.3 Isolation and genomic analysis of evolved bacteriophages

A total of 15 evolved phages were isolated after the evolution assay as indicated in the methods section and further characterized.

A BLASTn comparison was performed to identify the relationships between the genomes of the evolved and ancestral phages (Supplementary Table S3). Phages CUB_GE-MSSA31_R6, CUB_GE-MSSA31_R16, CUB_GE-MSSA31_R27-1, CUB_GE-MSSA31_R27-2, isolated on host strain GE-MSSA31, as well as phages CUB_BE-MRSA1_R7 and CUB_BE-MRSA1-R27 isolated on BE-MRSA1, were all related to the ancestral podovirus 44AHJD, with high sequence similarity and low number of SNPs (ranging from 0 to 4). Additionally, a similar antimicrobial activity was observed between the ancestral phage 44AHJD and its related evolved phages when tested by IMC (Supplementary Figure S4). These outcomes might suggest a lack of improvement of the phage descendants despite having undergone several rounds of evolution, possibly arising to some extent from a lack of genetic recombination with the myoviruses. Consequently, these 44AHJD phage mutants were excluded for further antibiofilm analysis.

The remaining phages isolated using host strains BE-MRSA1, GE-MRSA15 and MRSA-COL were all derived from the ancestral myoviruses. A comparative alignment showed a general trend in which phages exposed to fewer rounds of evolution (CUB_BE-MRSA1_R16, CUB_GE-MRSA15_R7 or CUB_MRSA-COL_R9) presented greater similarity to ancestral phages than those phages exposed to more rounds of evolution (CUB_GE-MRSA1_R30, CUB_GE-MRSA15_R14/R23 or CUB_MRSA-COL_R20/R23). Evolved phages isolated from host strains GE-MRSA15 and MRSA-COL were selected for further characterization, as they reached the highest bacterial reduction when tested in monotherapy against their respective host strain. Supplementary Table S4 shows the punctual missense mutations in the structural characterized proteins of evolved phages compared to their likely ancestor.

Most identified mutations in the characterized proteins with known function occurred in tail proteins such as in Ig-like domain containing proteins (Supplementary Table S5). Evolved phage CUB_MRSA-COL_R23 was the only phage with evidenced mutations in tail-type lysozymes when compared to ancestral phage ISP. Moreover, phage CUB_GE-MRSA15_R23 had several mutations in putative tail morphogenic protein F compared to ancestral phage M.

### 3.4 Host range analysis of ancestral and evolved phages

Fifty-one *S. aureus* strains (70.83%) were susceptible to at least one of the five ancestral phages (from which eight strains were susceptible to all five), whereas 21 strains (29.17%) within the collection did not show susceptibility to any ancestral phage (Supplementary Table S2). Among the 30 strains displaying methicillin resistance, 23 could be lysed by at least one of the ancestral phages.

As for the evolved phages, 69 bacterial strains (95.83%) were susceptible to at least one evolved phage, while only three (4.17%) strains showed resistance to all evolved phages, being also resistant to the ancestral phages. Out of the 30 methicillin resistant strains, 28 could be lysed by at least one of the evolved phages. Notably, evolved phages overcame the bacterial resistance observed with the ancestral phages. Two bacterial strains showed susceptibility to all evolved and ancestral phages.

Among the ancestral phages, phage ISP showed the broadest host range (44.44%), whereas phage CUB-B showed the narrowest host range (26.39%). On the other side, the evolved phage CUB_MRSA-COL_R9 could infect the largest number (83.33%) of the strains in the collection, in comparison to evolved phage CUB_BE-MRSA1_R7 infecting the lowest number (37.5%).

### 3.5 Antimicrobial efficacy of evolved bacteriophages evaluated by isothermal microcalorimetry

**Figure 3:**
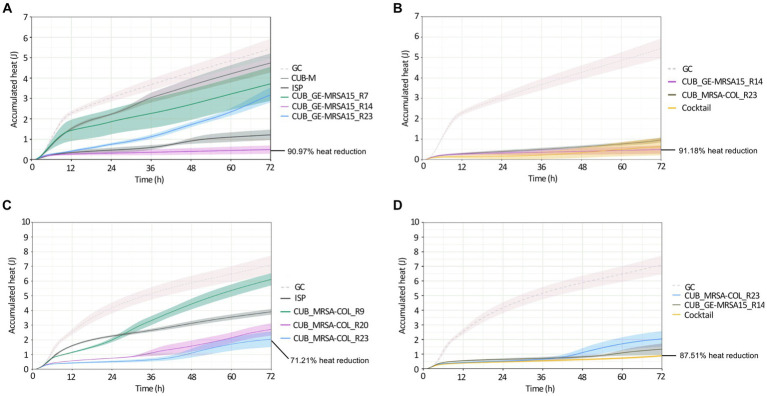
Calorimetric heat curves of biofilms exposed to evolved and ancestral phages. Each curve shows the heat (J) produced over time by viable bacteria in the biofilm over 72 h monitoring. **(A)** GE-MRSA15 biofilm exposed to ancestral (CUB-M and ISP) and evolved (CUB_GE-MRSA15_R7/R14/R23) phages. **(B)** GE-MRSA15 biofilm exposed to evolved phages CUB_GE-MRSA15_R14 and CUB_MRSA-COL_R23 alone or combined 1:1 as a phage cocktail. **(C)** MRSA-COL biofilm exposed to ancestral (ISP) and evolved (CUB_MRSA-COL_R9/R20/R23) phages. **(D)** MRSA-COL biofilm exposed to evolved phages CUB_GE-MRSA15_R14 and CUB_MRSA-COL_R23 alone or combined 1:1 as a phage cocktail. GC corresponds to the growth control sample not exposed to phages. Data are expressed as mean ± SE from three biologial replicates. Plots were prepared using RStudio [30]. Solid lines represent the mean and corresponding shaded regions the standard error.

IMC was used for the evaluation of the antimicrobial activity of evolved phages compared to the ancestral phages (Figure 3). Biofilms of *S. aureus* GE-MRSA15 and MRSA-COL were exposed to evolved phages CUB_GE-MRSA15_R7, CUB_GE-MRSA15_R14, CUB_GE-MRSA15_R23 and ancestral phages ISP and CUB-M (in the case of GE-MRSA15) or to CUB_MRSA-COL_R9, CUB_MRSA-COL_R20 and CUB_MRSA-COL_R23 (in the case of MRSA-COL), as well as to the ancestral phage ISP. Based on the observed results, the efficacy of a phage cocktail consisting of equal amounts of the evolved phages showing the highest antimicrobial activity was further investigated.

Panel A in Figure 3 shows the results of exposure of strain GE-MRSA15 to ancestral and evolved phages, while panel B shows the results of exposure of the same strain GE-MRSA15 to the cocktail of two evolved phages compared to the effect of each of these phages individually. Exposure of GE-MRSA15 to phage CUB-M showed a slight initial decrease in heat production compared to the untreated sample (growth control), but overall, no remarkable antimicrobial effect was observed during the whole monitoring period, reaching heat values after 72 h comparable to those of the growth control. On the contrary, phage ISP had a stronger antimicrobial effect, showing a remarkable decrease in total heat production of 84.71% up to 72 h. Regarding the antimicrobial activity of the evolved phages, phage CUB_GE-MRSA15_R14 displayed high heat suppression, with a 90.97% of total heat reduction (at 72 h) compared to the growth control, whereas phage CUB_GE-MRSA15_R7 had an effect (38.65%) similar to that of the ancestral phage CUB-M (26.56%). Phage CUB_GE-MRSA15_R23 showed a more progressive effect, where higher suppression could be observed at early times (75.20% at 24 h, *p* = <0.0001) to end up with heat values comparable to those of phage CUB_GE-MRSA15_R7 at the end of the monitoring time (41.77% at 72 h).

The phage cocktail combining evolved phages CUB_GE-MRSA15_R14 and CUB_MRSA-COL_R23 showed a high antimicrobial activity on time points 24 h (94.67%, *p* = <0.0001), and slightly decreased at 48 h (93.74%) and 72 h (91.18%).

Panel C in Figure 3 shows the results of exposure of strain MRSA-COL to ancestral and evolved phages, while panel D shows the results of exposure of the same strain MRSA-COL to the cocktail of two evolved phages compared to the effect of each of these phages individually. Heat curves obtained from the co-incubation of MRSA-COL biofilm with ancestral phage ISP revealed a moderate antimicrobial effect (44.99% of heat reduction, *p* = 0.0029) up to 72 h monitoring. When analyzing the outcomes of co-incubating biofilms with the evolved phages, we could observe that both phages isolated at latter rounds of evolution (CUB_MRSA-COL_R20 and CUB_MRSA-COL_R23) showed a higher antimicrobial effect (61.79 and 71.21% respectively) than the phage isolated at an earlier round (CUB_MRSA-COL_R9 with 13.90%) and the ancestral phage ISP. The phage cocktail showed an enhanced antimicrobial effect (87.51% *p* = <0.0001) compared to each phage alone.

### 3.6 Antibiofilm efficacy of evolved bacteriophages evaluated by RT-qPCR

**Figure 4:**
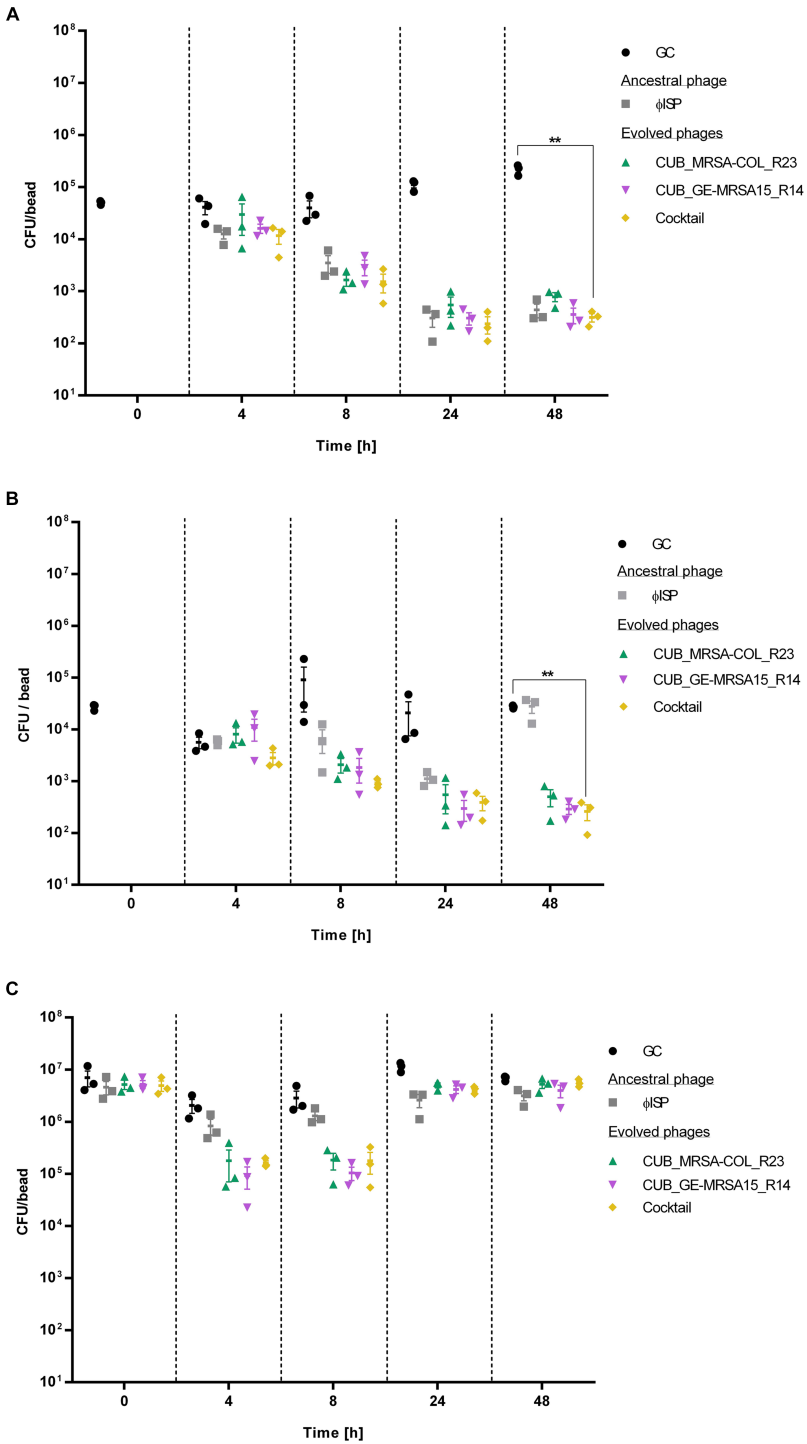
Bacterial cell count, expressed as colony-fomring-units per bead (CFU/bead), of **(A)** GE-MRSA15, **(B)** MRSA-COL and **(C)** MRSA-USA300 biofilms after exposure to phages (10^7^ PFU/mL) for 0 h, 4 h, 8 h, 24 h and 48 h determined by RT-qPCR. GC indicates the growth control sample not exposed to phages. Results were statistically analyzed using a student’s *t*-test using Welch correction with GraphPad Prism 8.0 (GraphPad Software, La Jolla, CA, USA).

The biofilm cell count reduction capabilities of evolved and ancestral phages was assessed by RT-qPCR following 4 h, 8 h, 24 h or 48 h of biofilm exposure to phages (Figure 4). Bacterial strains GE-MRSA15 (Figure 4A) and MRSA-COL (Figure 4B) as well as MRSA-USA300 (Figure 4C) (a strain not included in the evolution assay) were tested. MRSA-USA300 was included for the antibiofilm activity assessment to further explore whether the enhanced activity of the evolved phages extends beyond the strains to which they were exposed during evolution. Evolved phages CUB_MRSA-COL_R23 and CUB_GE-MRSA15_R14, alone or combined in a cocktail, were tested in this assay and compared to the activity of ancestral phage ISP.

The exposure of GE-MRSA15 biofilm to the phage cocktail at the timepoint of 4 h decreased the bacterial counts by 71.67% (<1 log) compared to the growth control. The phage cocktail’s antibiofilm activity increased progressively reaching a 96.17% (1.6 log) bacterial cell reduction after 8 h, of 99.79% after 24 h and of 99.86% (2.9 log) after 48 h (*p* = 0.0023). As for the biofilm exposure to single phage, the ancestral phage ISP showed the highest antibiofilm activity after 4 h of exposure reducing 69.42% (<1 log) the biofilm cell count, followed by evolved phage CUB_GE-MRSA15_R14 with a reduction of 60.68% (<1 log) and evolved phage CUB_MRSA-COL_R23 with a reduction of 27.72%. Between 8 and 24 h, all phages had a similar performance (>99%/ >2 log of reduction), including the phage cocktail. The antibiofilm activity slightly increased until the last measurement at 48 h. No regrowth of bacterial cells was observed during the evaluated timepoints, neither after the treatment with the ancestral nor with evolved phages.

Similarly, the phage cocktail showed the highest antibiofilm activity on MRSA-COL with a biofilm cell count reduction of 99.04% (2 log) (*p* = 0.0014) compared to the growth control after 48 h exposure. Regarding exposure to single phages, after 4 h, the antibiofilm activity displayed by ancestral phage ISP (62.58%/<1 log, *p* = 0.3209) outperformed that of the evolved phages CUB_MRSA-COL_R23 and CUB_GE-MRSA15_R14, with a difference in bacteria load reduction of 14.95 and 32.66%, respectively. All tested conditions, however, showed similar performance after 8 h of exposure (>90%/1 log of load reduction). When monitoring 24 h of exposure, evolved phages CUB_MRSA-COL_R23 and CUB_GE-MRSA15_R14, alone (97.69%, *p* = 0.2670 and 97.97%, *p* = 0.2629) and combined in a phage cocktail (99.00%/2 log *p* = 0.2644), displayed higher antibiofilm effect compared to ancestral phage ISP (92.66%/1 log *p* = 0.2769). At the last measured time point (48 h), evolved phages CUB_MRSA-COL_R23 and CUB_GE-MRSA15_R14 reached a reduction of 98.16% (1.9 log) (*p* = 0.0011) and 98.93% (2 log) (*p* = 0.0014), respectively. The biofilm exposed for 48 h to the ancestral phage presented a similar cell count as the growth control at that time point. No regrowth was evidenced with the evolved phages alone or combined in a phage cocktail.

Evolved phages, alone or combined, revealed a higher bacterial count reduction than the ancestral phage against MRSA-USA300. A 91.32% (<2 log) and 93.59% (<2 log) cell reduction were observed for phage CUB_MRSA-COL_R23 after 4 and 8 h exposure respectively, whereas phage CUB_GE-MRSA15_R14 reached a 95.46% (<2 log) and 96.35% (<2 log) cell reduction, but only after 4 and 8 h exposure, not 48 h. Notably, the reductions were greater than those observed with the ancestral phage ISP after 4 h (59.65%/<1 log) and 8 h (54.46%/<1 log) of exposure. In contrast to what was observed with the strains GE-MRSA15 and MRSA-COL, included in the evolution assay, after 24 h and 48 h of exposure to evolved phages, there is no remarkable differences in cell count between the treated strain MRSA-USA300 compared to the growth control, indicating a regrowth of the biofilm.

## Discussion

Developing new treatment strategies against biofilm-related infections is crucial, especially for the management of difficult to treat infections caused by multidrug resistant *S. aureus*. In this study, we implemented an evolutionary approach to enhance phage activity against pre-stablished *S. aureus* biofilms.

Having access to a diverse and clinically relevant panel of bacterial strains, covering a wide range of genomic and antibiotic resistance profiles, is of extreme value for the development of novel antimicrobial therapeutics (Galac et al., 2020; Lebreton et al., 2021). For our *in vitro* assay, we assembled a panel of 72 *S. aureus* strains, collected at four different European geographical locations and grouped into four clusters based on nucleotide similarity of the core genes, including a high proportion (40.28%) of strains displaying methicillin resistance. One of the strains was resistance to vancomycin, whereas 6 strains (8%) were resistant to rifampicin.

Phages have evolved alongside their bacterial hosts and often have mechanisms that allow them to infect their hosts also in the presence of biofilms (Matz, 2009). Previous studies have shown improvement of antimicrobial properties such as expanded host range (Akusobi et al., 2018; Sergueev et al., 2019; Sáez Moreno et al., 2021) or higher bacterial suppression (Akusobi et al., 2018; Borin et al., 2021) of evolved phages following directed evolution. We hypothesized that the antibiofilm performance of phages could also be enhanced by exposure of phages to a series of sequential infection cycles, or “phage passages.” Unlike the above-mentioned previous studies using directed phage evolution, which were conducted using planktonic bacteria, the goal of our work was to adapt phages especially toward biofilm of *S. aureus*.

The passage assay comprised three segments, each with 10 rounds of evolution. The phage mixture from the top-performing evolution round (the round in which the highest heat reduction was observed) from each segment, for each host bacteria, was used to isolate evolved phages. This way we aimed to compare the phage performance as a function of the number of passages to which they were subjected. During 30 rounds of directed evolution, we observed a progressive overall improvement in the activity of the phage mixture in inhibiting biofilm heat production over the whole experimental assay, with alternating rises and falls in activity at consecutive rounds of evolution. This positive trend might be due to an increased chance for random mutagenesis and recombination events leading to the accumulation of beneficial mutations when the phage undergoes a greater number of passages. Phages with beneficial mutations may therefore persist throughout the assay while phages with unfavorable mutations may perish in early rounds and not be present in later rounds. The absence of recombination events could be the reason why descendants of podovirus 44-AHJD show no improvement in their antimicrobial activity even after 27 rounds of evolution. Furthermore, only a slight improvement of the host range was evidenced going from a coverage of 40.38% of the ancestral phage performance to a 48–55% of coverage for the evolved phages. Strain GE-MRSA31’s evolved phages underwent four or less missense mutations compared to the ancestral phage. This might be in part due to a lack of recombination events between the podovirus and the myovirus. Previous research (Kornienko et al., 2020) suggests that the quick adsorption and shorter latent period of myoviruses likely outcompete podoviruses during co-incubation, resulting in lower concentrations along passages.

From the host range analysis, we could observe that 27% of strains in our *S. aureus* collection were not infected by any of the ancestral phages, while evolved phages were able to overcome most of the initial bacterial resistance, resulting in 95% of the bacterial strains being susceptible to at least one evolved phage. Sometimes phages from early rounds such as CUB_MRSA-COL_R9 had better host coverage (83.33%) than phages from later rounds such as CUB_MRSA-COL_R20 and CUB_MRSA-COL_R23 (48.61 and 59.72% respectively). In the same way, phages such as CUB_BE-MRSA1_R7 from early rounds had worse performance (37.5%) compared to phages from later rounds such as CUB_BE-MRSA1_R26 with a coverage of 66.67%. Therefore, we cannot conclude that there’s a correlation between more exposure to passages and broader coverage of strains.

The antimicrobial activity analysis evaluated by IMC revealed that evolved phages isolated after a higher number of evolution rounds in general exhibited greater heat reduction compared to those isolated after a lower number of rounds. However, exposure of phages to more rounds of evolution did not always result in enhanced performance. For example, when considering phage CUB_GE-MRSA15_R14, isolated from round 14 on the strain GE-MRSA15, it outperformed phage CUB_GE-MRSA15_R23 obtained from round 23 on the same strain. Looking at the genome sequences, phage CUB_GE-MRSA15_R23 possesses 24 extra missense mutations compared to phage CUB_GE-MRSA15_R14, which could be the reason for the different performance. Esvelt et al. (2011) describes that a directed evolution allows the creation of mutations using distinct trajectories, which might converge and end up with proteins with the same function. In our case, we expected that the increase in biofilm activity would be a consequence of mutations in membrane and enzymatic proteins, which would allow phages to be more virulent and effective. As shown in Supplementary Table S5, the mutations that could be tracked occurred on structural wall proteins. The lack of function knowledge hinders the possibility of correlating mutation in proteins with their function. Further functional genomic experiments are essential to find out if the mutations of other hypothetical proteins are responsible for the observed antimicrobial enhancement.

Using RT-qPCR, we accurately assessed the antibiofilm degradation ability of evolved phages and compared it to the ancestral phage as well as to the performance as a phage cocktail. Formulating phage cocktails is crucial as they combine multiple phages to broaden bacterial coverage (Eskenazi et al., 2022), enhance treatment efficacy, mitigate resistance and target biofilms (Kifelew et al., 2020). Considering this, our phage cocktail was formulated selecting the two phages with highest performance as observed by IMC assays and covers 65.28% of the bacterial biobank in terms of host range, being superior to each phage alone. The phage cocktail showed a strong biofilm degradation capability against GE-MRSA15 and MRSA-COL strains (included in the evolution assay) with no biofilm regrowth up to 48 h. In the case of strain MRSA-USA300 (not included in the evolution assay) evolved phages had a higher antibiofilm efficacy at initial incubation time (up to 8 h) compared to ancestral phage ISP, which could indicate the possible benefit of repeated doses of phages in clinical application or staggered combination with other antimicrobials. However, after 48 h of monitoring, the evolved phages did not exhibit a superior effect when compared to the ancestral phages, which might be due to resistance emergence to phages.

In contrast to genetic engineering methods (Chen et al., 2019), where mutation can be exact, directed evolution based on serial passages is limited by the fact that mutations occur randomly. The benefits of directed evolution are its simplicity, effectiveness, and safety. This strategy highlights the potential of phage passages to overcome the difficulties caused by *S. aureus* infections linked with biofilms. Our study and other studies show that phage passages are efficient in enhancing lytic activity against *S. aureus*. According to Sáez Moreno et al. (2021) following passages, phages exhibited a better infectivity toward planktonic cells and enhanced binding affinity. Analogous results were also shown in a previous study with *P. aeruginosa* biofilms (Kunisch et al., 2023a). Although there’s little to no eradication of biofilm cell numbers depending on the *S. aureus* isolate. Various strategies involving a combination of evolved phages and antibiotics can be formulated to address biofilm defenses (Doub et al., 2021).

In conclusion, this study underscores the effectiveness of a directed evolution approach through sequential phage passages. The observed progressive improvement in phage performance, couple with the expanded host range and superior antibiofilm efficacy of the formulated phage cocktail, highlights the potential of this strategy as a valuable tool in combating multidrug-resistant strains, particularly those associated with biofilms, offering a promising avenue for future antimicrobial therapeutics. Further research, including *in vivo* studies, is crucial to validate these findings and advance phage therapy as a viable treatment option for biofilm-related infections.

## Data availability statement

The data presented in the study are deposited in the NCBI repository, under name BioProject: PRJNA952137.

## Author contributions

LP: Writing – review & editing, Writing – original draft. JW: Writing – review & editing, Writing – original draft. DH: Writing – review & editing, Writing – original draft. AA: Writing – review & editing, Writing – original draft. RL: Writing – review & editing, Writing – original draft. AT: Writing – review & editing, Writing – original draft. MG: Writing – review & editing, Writing – original draft.
