# Supplementary material for: Targeted enhancement of bacteriophage activity against antibiotic-resistant Staphylococcus aureus biofilms through an evolutionary assay
Source: Front Microbiol. 2024 Jul 8;15:1372325. doi: 10.3389/fmicb.2024.1372325 (PMC11260789; doi:10.3389/fmicb.2024.1372325)
Supplement: Supplementary file 1 [file Data_Sheet_1.PDF]

Targeted enhancement of bacteriophage activity against antibiotic-resistant *Staphylococcus aureus* biofilms through an evolutionary assay

L. Ponce Benavente, J. Wagemans, D. Hinkel, A. Aguerri Lajusticia, R. Lavigne, A. Trampuz, M. Gonzalez Moreno

Supplementary data

Figures

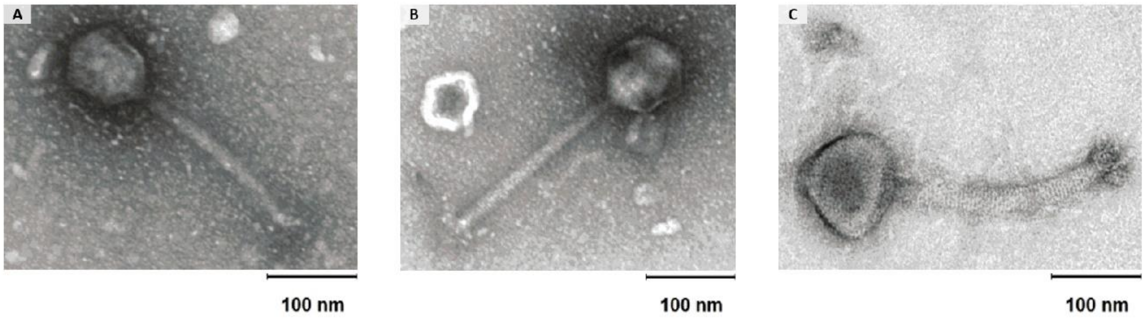

Figure S1. Transmission electron microscopy images of the three isolated ancestral bacteriophages CUB-A (A), CUB-B (B) and CUB-M (C).

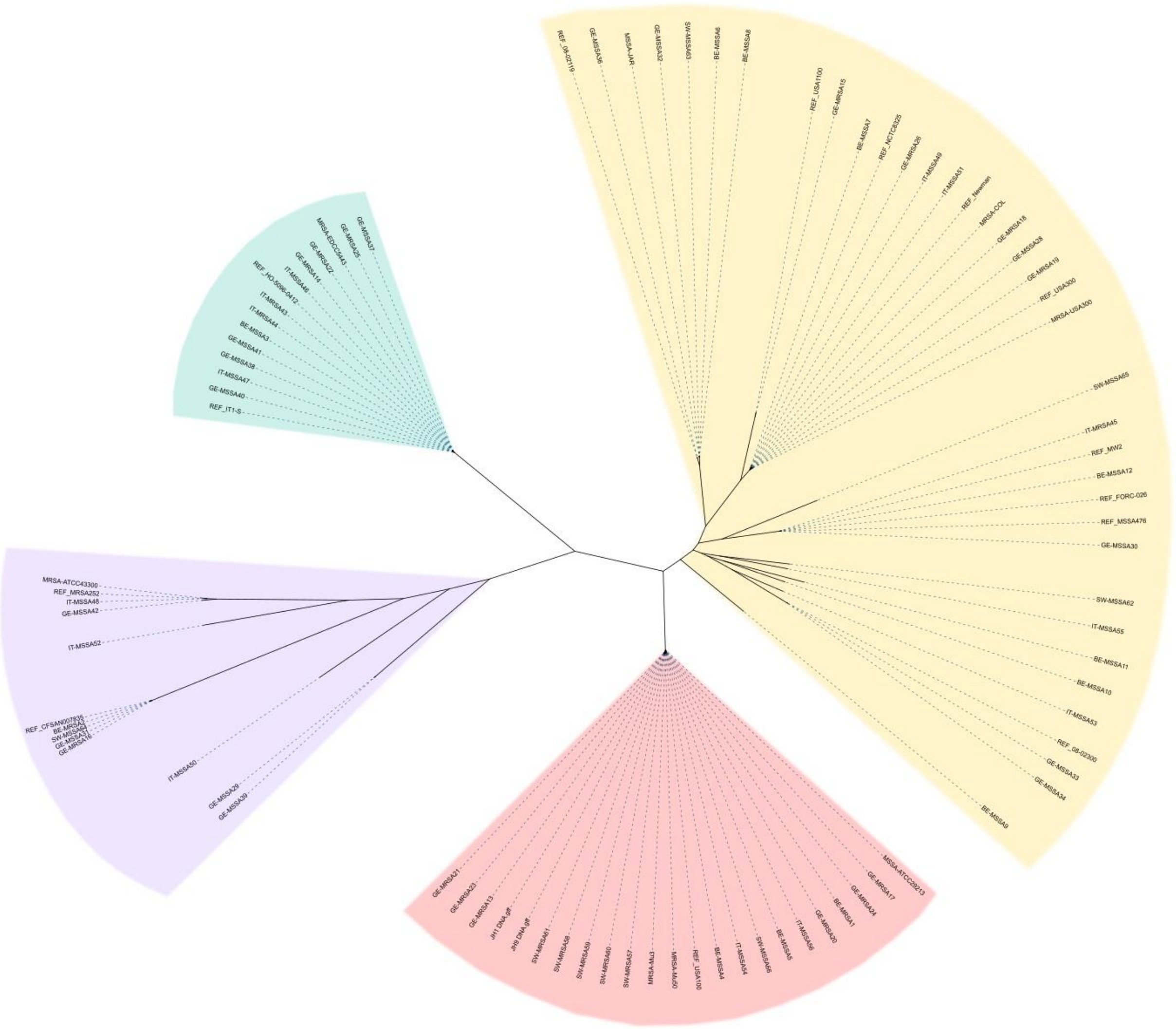

Figure S2. Phylogenetic tree of all *S. aureus* strains in the collection spanning four genomic clusters. Cluster 1 (yellow) comprising 38.89% of strains, Cluster 2 (red) with 27.78% of strains, Cluster 3 (green) with 18.06% of strains and Cluster 4 (purple) with 15.28% of strains.

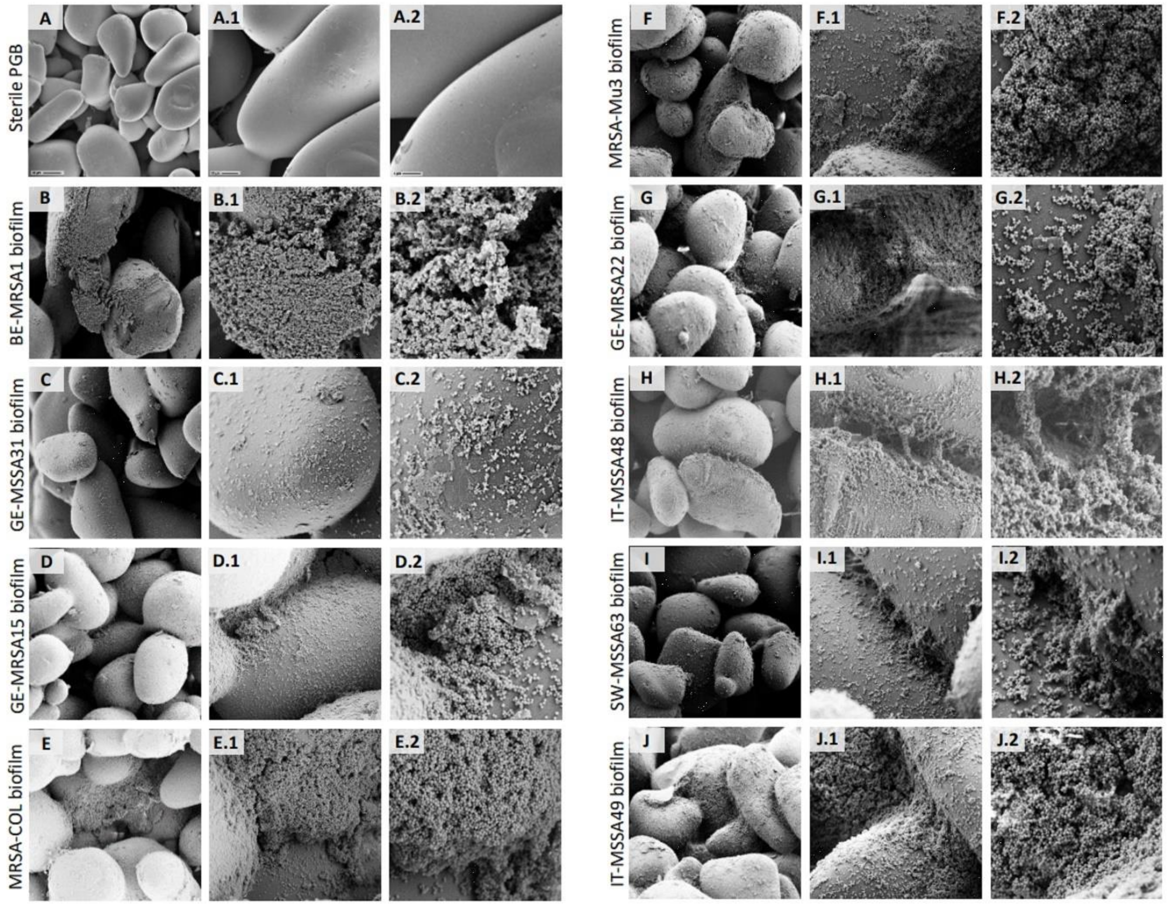

Figure S3. Scanning electron microscopy images of the pre-stablished 24-h-biofilm of *S. aureus* strains included in the evolution assay and a sterile porous glass bead (A/A.1/A.2) displayed at three different magnifications (left column – Mag 100 X; middle column – Mag 20 500 X; right column – Mag 5000 X).

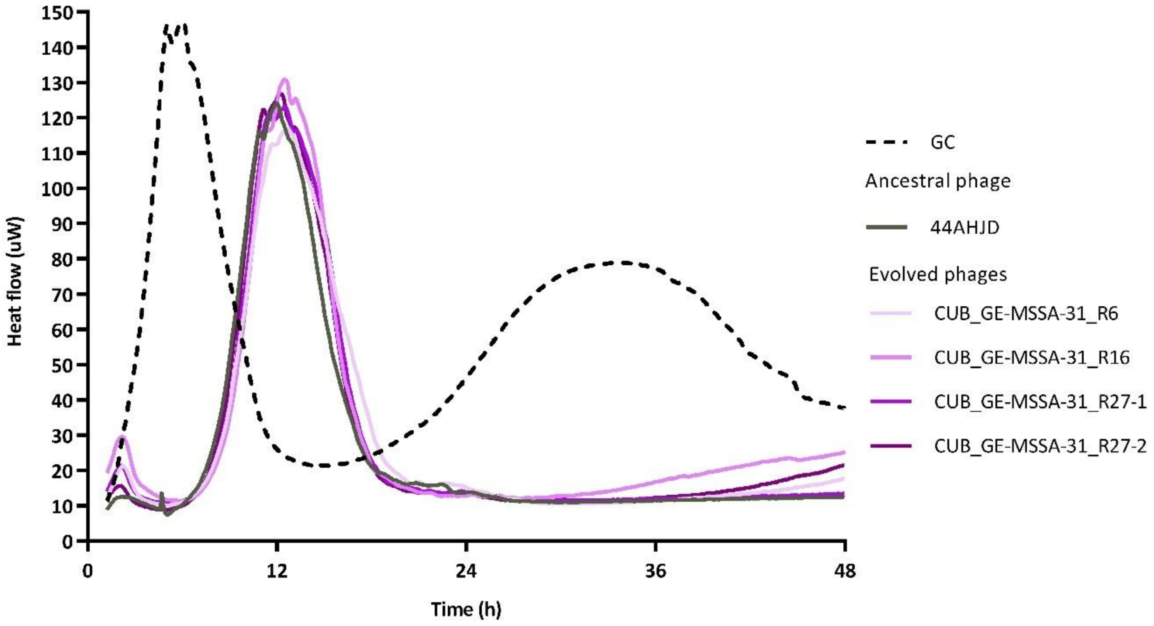

Figure S4. Heat flow curve (µW) of GE-MRSA31 biofilm exposed to ancestral and evolved phages during 48 hours. GC, growth control.

Tables

Table S1. Heat map representing antibiotic susceptibility of the *S. aureus* collection (n = 72).

Bacterial strains	Antibiotics																											
	Ampicilin	Piperacillin	AMP/SUL	PIP/TAZ	Imipenem	Meropenem	Cefazolin	Cefuroxim	Ceftriaxon	Gentamicin	Tobramycin	Doxycyclin	Cotrimoxazol	Ciprofloxacin	Penicilin	Oxacilin	Erythromycin	Clindamicin	Vancomycin	Rifampicin	Linezolid	Fosfomycin	Tigecyclin	Levofloxacin	Fusidinsäure	Mupirocin	Teicoplanin	Daptomycin
BE-MRSA1	R	R	R	R	R	R	R	R	R	S	S	S	S	R	R	R	S	S	S	S	S	S	S	R	R	S	S	S
BE-MRSA2	R	R	R	R	R	R	R	R	R	S	S	S	S	R	R	R	R	R	S	S	S	S	S	R	S	S	S	S
BE-MSSA3	R	R	S	S	S	S	S	S	I	S	S	S	S	I	R	S	S	S	S	S	S	S	S	I	S	S	S	S
BE-MSSA4			S	S	S	S	S	S	I	S	S	S	S	I		S	S	S	S	S	S	S	S	I	S	S	S	S
BE-MSSA5			S	S	S	S	S	S	I	S	S	S	S	R		S	R	R	S	S	S	S	S	I	S	S	S	S
BE-MSSA6	R	R	S	S	S	S	S	S	I	S	S	S	S	I	R	S	S	S	S	S	S	S	S	I	S	S	S	S
BE-MSSA7	R	R	S	S	S	S	S	S	I	S	S	S	S	I	R	S	S	S	S	S	S	S	S	I	S	S	S	S
BE-MSSA8	R	R	S	S	S	S	S	S	I	S	S	S	S	I	R	S	S	S	S	S	S	S	S	I	S	S	S	S
BE-MSSA9	R	R	S	S	S	S	S	S	I	S	S	S	S	I	R	S	S	S	S	S	S	S	S	I	S	S	S	S
BE-MSSA10	R	R	S	S	S	S	S	S	I	S	S	S	S	I	R	S	S	S	S	S	S	S	S	I	S	S	S	S
BE-MSSA11	R	R	S	S	S	S	S	S	I	S	S	S	S	I	R	S	S	S	S	S	S	S	S	I	S	S	S	S
BE-MSSA12	R	R	S	S	S	S	S	S	I	S	S	S	S	I	R	S	S	S	S	S	S	S	S	I	S	S	S	S
GE-MRSA13	R	R	R	R	R	R	R	R	R	S	S	S	S	R	R	R	R	R	S	S	S	S	S	R	R	S	S	S
GE-MRSA14	R	R	R	R	R	R	R	R	R	S	S	S	S	R	R	R	S	S	S	S	S	S	S	R	S	S	S	S
GE-MRSA15	R	R	R	R	R	R	R	R	R	R	R	R	R	R	R	R	R	R	S	R	S	S	S	R	R	S	S	S
GE-MRSA16	R	R	R	R	R	R	R	R	R	S	S	R	S	I	R	R	S	S	S	S	S	S	S	I	S	S	S	S
GE-MRSA17	R	R	R	R	R	R	R	R	R	S	S	S	S	R	R	R	S	S	S	S	S	S	S	R	S	S	S	S
GE-MRSA18	R	R	R	R	R	R	R	R	R	S	S	S	S	R	R	R	S	S	S	S	S	S	S	R	S	S	S	S
GE-MRSA19	R	R	R	R	R	R	R	R	R	S	S	S	S	R	R	R	S	S	S	S	S	S	S	R	S	S	S	S
GE-MRSA20	R	R	R	R	R	R	R	R	R	S	S	R	S	I	R	R	R	R	S	S	S	S	S	I	S	S	S	S
GE-MRSA21	R	R	R	R	R	R	R	R	R	S	S	S	S	R	R	R	R	R	S	S	S	S	S	R	S	S	S	R
GE-MRSA22	R	R	R	R	R	R	R	R	R	S		S	S		R	R	I	R	S	R	S	R	S	R	R	I	S	S
GE-MRSA23	R	R	R	R	R	R	R	R	R	S	S	S	S	R	R	R	R	R	S	S	S	S	S	R	S	S	S	S
GE-MRSA24	R	R	R	R	R	R	R	R	R	S	S	S	S	I	R	R	S	S	S	S	S	S	S	I	S	S	S	S
GE-MRSA25	R	R	R	R	R	R	R	R	R	S	S	S	S	R	R	R	R	R	S	S	S	S	S	R	S	S	S	S
GE-MRSA26	R	R	R	R	R	R	R	R	R	S	S	S	S	I	R	R	S	S	S	S	S	S	S	I	R	S	S	S
GE-MSSA28			S	S	S	S	S	S	S	S	S	S		S		S	S	S	S	S	S	S	S	S	S	S	S	S
GE-MSSA29	S	R	R	S	S	S	S	S	S	S	S	S		S	R	S	S	S	S	S	S	S	S	S	S	S	S	S
GE-MSSA30	R	R	S	S	S	S	S	S	S	S	S	S		S	R	S	S	S	S	S	S	S	S	S	R	S	S	S
GE-MSSA31	R	R	S	S	S	S	S	S	S	S	S	R		S	R	S	R	S	S	R	S	S	S	S	S	S	S	S
GE-MSSA32	R	R	S	S	S	S	S	S	S	S	S	S		S	R	S	S	S	S	S	S	S	S	S	S	S	S	S
GE-MSSA33	R	R	S	S	S	S	S	S	S	S	S	S		S	S	S	S	S	S	S	S	S	S	S	S	S	S	S
GE-MSSA34	R	R	S	S			S	S	S	S	S	S		S	R	S	R	R	S	S	S	S	S	S	S	S	S	S
GE-MSSA36	R	R	S	S	S	S	S	S	S	R	R	S		S	R	S	S	S	S	S	S	S	S	S	S	S	S	S
GE-MSSA37	R	R	S	S	S		S	S	S	S	S	S		R	R	S	S	S	S	S	S	S	S	R	S	S	S	S
GE-MSSA38	R	R	S	S	S	S	S	S	I	S	S	S	S	I	R	S	S	S	S	S	S	S	S	I	S	S	S	S
GE-MSSA39	R	R	S	S	S	S	S	S	I	S	S	S	S	I	R	S	S	S	S	S	S	S	S	I	S	S	S	S
GE-MSSA40	R	R	S	S	S	S	S	S	I	S	S	S	S	R	R	S	R	R	S	S	S	S	S	I	S	S	S	S
GE-MSSA41	R	R	S	S	S	S	S	S	I	S	S	S	S	I	R	S	S	S	S	S	S	S	S	I	S	S	S	S
GE-MSSA42	R	R	S	S	S	S	S	S	I	S	S	S	S	I	R	S	S	S	S	S	S	S	S	I	S	S	S	S
IT-MRSA43	R	R	R	R	R	R	R	R	R	S	S	S	S	R	R	R	R	R	S	S	S	S	S	R	S	S	S	S
IT-MRSA44	R	R	R	R	R	R	R	R	R	S	S	S	S	R	R	R	S	S	S	S	S	S	S	R	S	S	S	S
IT-MRSA45	R	R	R	R	R	R	R	R	R	S	S	R	S	I	R	R	R	R	S	S	S	S	S	I	S	S	S	S
IT-MSSA46																												
IT-MSSA47	R	R	S	S	S	S	S	S	I	S	S	S	S	I	R	S	S	S	S	S	S	S	S	I	S	S	S	S
IT-MSSA48										S		S	S		R	S	I	I	S	R	S	S	S	I	S	S	S	S
IT-MSSA49	R	R	S	S	S	S	S	S	I	S	S	S	S	I	R	S	S	S	S	R	S	S	S	I	S	S	S	S
IT-MSSA50	R	R	S	S	S	S	S	S	I	S	S	S	S	I	R	S	S	S	S	S	S	S	S	I	S	S	S	S
IT-MSSA51	R	R	S	S	S	S	S	S	I	S	S	S	S	I	R	S	S	S	S	S	S	S	S	I	S	S	S	S

Table S1. Continuation

Bacterial strains	Ampicilin	Piperacillin	AMP/SUL	PIP/TAZ	Imipenem	Meropenem	Cefazolin	Cefuroxim	Ceftriaxon	Gentamicin	Tobramycin	Doxycyclin	Cotrimoxazol	Ciprofloxacin	Penicilin	Oxacilin	Erythromycin	Clindamicin	Vancomycin	Rifampicin	Linezolid	Fosfomycin	Tigecyclin	Levofloxacin	Fusidinsäure	Mupirocin	Teicoplanin	Daptomycin
	**Antibiotics**																											
IT-MSSA53	R	R	S	S	S	S	S	S	I	S	S	S	S	I	R	S	S	S	S	S	S	S	S	I	S	S	S	S
IT-MSSA54	R	R	S	S	S	S	S	S	I	S	S	S	S	I	R	S	S	S	S	S	S	R	S	I	S	S	S	S
IT-MSSA55	R	R	S	S	S	S	S	S	I	R	R	S	S	R	R	S	S	S	S	S	S	S	S	I	S	S	S	S
IT-MSSA56	R	R	S	S	S	S	S	S	I	S	S	S	S	I	R	S	S	S	S	S	S	R	S	I	S	S	S	S
SW-MRSA57	R	R	R	R	R	R	R	R	R	R	R	S	S	R	R	R	R	R	S	S	S	S	S	R	S	S	S	S
SW-MRSA58	R	R	R	R	R	R	R	R	R	R	R	S	S	R	R	R	R	R	S	S	S	S	S	R	S	I	S	S
SW-MRSA59	R	R	R	R	R	R	R	R	R	R	R	S	S	R	R	R	R	R	S	S	S	S	S	R	S	S	S	S
SW-MRSA60	R	R	R	R	R	R	R	R	R	R	R	S	S	R	R	R	R	R	S	S	S	S	S	R	S	I	S	S
SW-MRSA61	R	R	R	R	R	R	R	R	R	R	R	S	S	R	R	R	R	R	S	S	S	S	S	R	S	I	S	S
SW-MSSA62	R	R	S	S	S	S	S	S	I	S	S	S	S	I	R	S	S	S	S	S	S	S	S	I	S	S	S	S
SW-MSSA63			S	S	S	S	S	S	I	S	S	S	S	I		S	S	S	S	S	S	R	S	I	S			S
SW-MSSA64	R	R	S	S	S	S	S	S	I	S	S	S	S	I	R	S	S	S	S	S	S	S	S	I	S	S	S	S
SW-MSSA65	R	R	S	S	S	S	S	S	I	S	S	S	S	I	R	S	S	S	S	S	S	S	S	I	S	S	S	S
SW-MSSA66			S	S	S	S	S	S	I	S	S	S	S	I		S	S	S	S	S	S	S	S	I	S	S	S	S
MRSA-ATCC43300	R	R	R	R	R	R	R	R	R	R	R	S	S	I	R	R	R	R	S	S	S	S	S	I	S	S	S	S
MRSA-Mu3	R	R	R	R	R	R	R	R	R	R	R	R	S	R	R	R	R	R	S	S	S	R	S	R	S	S	S	S
MRSA-Mu50										R		R	S		R	R	R	R	R	R	S	R	S	R	S	S	S	R
MRSA-COL	R	R	R	R	R	R	R	R	R	S	S	R	S	I	R	R	S	S	S	S	S	S	S	I	S	S	S	S
MRSA-USA300			S	S	S	S	S	S	I	S	S	S	S	R		S	S	S	S	S	S	S	S	R	S	S	S	S
MRSA-EDCC5443	R	R	R	R	R	R	R	R	R	S	S	S	S	R	R	R	R	R	S	S	S	S	S	R	S	S	S	S
MSSA-ATCC29213	R	R	S	S	S	S	S	S	I	S	S	S	S	I	R	S	S	S	S	S	S	S	S	I	S	S	S	S
MSSA-JAR	R	R	S	S	S	S	S	S	I	S	S	S	S	I	R	S	S	S	S	S	S	S	S	I	S	S	S	S

Green squares indicate susceptibility (S), red indicates resistance (R), yellow indicates susceptibility at higher doses (I) and gray squares indicate no susceptibility tested.

AMP/SUL: ampicilin/sulbactan; PIP/TAZ: piperacilin/tazobactam.

Table S2. Host range of ancestral and evolved phages against the collection of *S. aureus* strains (n = 72).

	Ancestral phages					Evolved phages														
Bacterial strains	CUB_M	CUB_A	CUB_B	ISP	44-AHJD	CUB_GE-MRSA15_R7	CUB_GE-MRSA15_R14	CUB_GE-MRSA15_R23	CUB_BE-MRSA1_R7	CUB_BE-MRSA1_R16	CUB_BE-MRSA1_R26	CUB_BE-MRSA1_R30	CUB_GE-MSSA31_R6	CUB_GE-MSSA31_R16	CUB_GE-MSSA31_R27-1	CUB_GE-MSSA31_R27-2	CUB_MRSA-COL_R9-1	CUB_MRSA-COL_R9-2	CUB_MRSA-COL_R20	CUB_MRSA-COL_R23
BE-MRSA1																				
BE-MRSA2																				
BE-MSSA3																				
BE-MSSA4																				
BE-MSSA5																				
BE-MSSA6																				
BE-MSSA7																				
BE-MSSA8																				
BE-MSSA9																				
BE-MSSA10																				
BE-MSSA11																				
BE-MSSA12																				
GE-MRSA13																				
GE-MRSA14																				
GE-MRSA15																				
GE-MRSA16																				
GE-MRSA17																				
GE-MRSA18																				
GE-MRSA19																				
GE-MRSA20																				
GE-MRSA21																				
GE-MRSA22																				
GE-MRSA23																				
GE-MRSA24																				
GE-MRSA25																				
GE-MRSA26																				
GE-MSSA28																				
GE-MSSA29																				
GE-MSSA30																				
GE-MSSA31																				
GE-MSSA32																				
GE-MSSA33																				
GE-MSSA34																				
GE-MSSA36																				
GE-MSSA37																				
GE-MSSA38																				
GE-MSSA39																				
GE-MSSA40																				
GE-MSSA41																				
GE-MSSA42																				
IT-MRSA43																				
IT-MRSA44																				
IT-MRSA45																				
IT-MSSA46																				
IT-MSSA47																				
IT-MSSA48																				
IT-MSSA49																				
IT-MSSA50																				
IT-MSSA51																				
IT-MSSA52																				
IT-MSSA53																				
IT-MSSA54																				
IT-MSSA55																				
IT-MSSA56																				

Table S2. Continuation

Bacterial strains	Ancestral phages					Evolved phages														
	CUB_M	CUB_A	CUB_B	ISP	44-AHJD	CUB_GE-MRSA15_R7	CUB_GE-MRSA15_R14	CUB_GE-MRSA15_R23	CUB_BE-MRSA1_R7	CUB_BE-MRSA1_R16	CUB_BE-MRSA1_R26	CUB_BE-MRSA1_R30	CUB_GE-MSSA31_R6	CUB_GE-MSSA31_R16	CUB_GE-MSSA31_R27-1	CUB_GE-MSSA31_R27-2	CUB_MRSA-COL_R9-1	CUB_MRSA-COL_R9-2	CUB_MRSA-COL_R20	CUB_MRSA-COL_R23
SW-MRSA57	Gray	Gray	Gray	Gray	Green	Green	Green	Green	Gray	Green	Green	Green	Green	Green	Green	Green	Green	Green	Green	Green
SW-MRSA58	Gray	Gray	Gray	Green	Gray	Green	Green	Green	Gray	Green	Gray	Green	Gray	Gray	Gray	Gray	Green	Green	Green	Green
SW-MRSA59	Gray	Gray	Gray	Gray	Green	Green	Gray	Gray	Gray	Gray	Green	Gray	Green	Green	Green	Green	Gray	Green	Gray	Gray
SW-MRSA60	Gray	Gray	Gray	Gray	Gray	Gray	Gray	Gray	Gray	Gray	Gray	Gray	Gray	Gray	Gray	Gray	Gray	Gray	Gray	Gray
SW-MRSA61	Gray	Gray	Gray	Gray	Green	Green	Green	Gray	Green	Green	Green	Gray	Green	Green	Green	Green	Green	Green	Gray	Green
SW-MSSA62	Green	Green	Green	Green	Green	Green	Green	Green	Green	Green	Green	Green	Green	Green	Green	Green	Green	Green	Gray	Gray
SW-MSSA63	Gray	Gray	Gray	Gray	Gray	Gray	Gray	Gray	Gray	Gray	Gray	Gray	Gray	Gray	Gray	Gray	Gray	Gray	Gray	Gray
SW-MSSA64	Green	Green	Green	Green	Green	Green	Green	Green	Green	Green	Green	Green	Green	Green	Green	Green	Green	Green	Green	Green
SW-MSSA65	Gray	Green	Green	Gray	Gray	Green	Green	Gray	Gray	Green	Gray	Green	Green	Gray	Gray	Gray	Green	Green	Gray	Green
SW-MSSA66	Gray	Gray	Gray	Green	Gray	Green	Green	Green	Green	Green	Green	Green	Gray	Gray	Gray	Gray	Green	Green	Green	Green
MRSA-ATCC43300	Green	Gray	Gray	Green	Green	Green	Green	Green	Gray	Gray	Green	Green	Green	Green	Green	Green	Green	Green	Green	Green
MRSA-Mu3	Green	Gray	Gray	Green	Gray	Gray	Gray	Gray	Gray	Gray	Gray	Gray	Gray	Gray	Gray	Gray	Gray	Green	Gray	Gray
MRSA-Mu50	Gray	Gray	Gray	Gray	Gray	Gray	Gray	Gray	Gray	Gray	Gray	Gray	Gray	Gray	Gray	Gray	Gray	Gray	Gray	Gray
MRSA-COL	Gray	Green	Gray	Green	Gray	Green	Green	Green	Gray	Green	Gray	Green	Gray	Gray	Gray	Gray	Green	Green	Green	Green
MRSA-USA300	Green	Gray	Gray	Green	Gray	Green	Green	Green	Gray	Green	Gray	Green	Gray	Gray	Gray	Gray	Green	Green	Green	Green
MRSA-EDCC5443	Gray	Gray	Gray	Green	Green	Gray	Gray	Gray	Gray	Gray	Green	Gray	Green	Green	Green	Green	Gray	Green	Gray	Gray
MSSA-ATCC29213	Gray	Gray	Gray	Gray	Green	Green	Green	Green	Green	Green	Green	Green	Green	Green	Green	Green	Gray	Green	Green	Green
MSSA-JAR	Green	Gray	Gray	Green	Gray	Green	Green	Gray	Gray	Green	Green	Green	Gray	Gray	Gray	Gray	Green	Green	Gray	Green

Green squares indicate susceptibility and gray squares indicate non-susceptibility of the bacterial strain to the tested phages.

Table S3. Genomic relationships between the genomes of the evolved and ancestral phages. QC: queue coverage, PI: porcentage of identity

Phages	CUB_A	CUB_B	CUB_M	ISP	44-AHJD	CUB_BE-MRSA1_R7	CUB_BE-MRSA1_R16	CUB_BE-MRSA1_R26	CUB_BE-MRSA1_R30	CUB_GE-MRSA15_R7	CUB_GE-MRSA15_R14	CUB_GE-MRSA15_R23	CUB_MRSA-COL _R9	CUB_MRSA-COL_R20	CUB_MRSA-COL _R23	CUB_GE-MSSA31_R6	CUB_GE-MSSA31_R16	CUB_GE-MSSA31_R27-1
CUB_BE-MRSA1_R7	QC(0%) PI(0)	QC(0%) PI(0)	QC(0%) PI(0)	QC(0%) PI(0)	QC(98%) PI(99,98)													
CUB_BE-MRSA1_R16	QC(87%) PI(97,11)	QC(86%) PI(96,69)	QC(88%) PI(99,35)	QC(88%) PI(99,31)	QC(0%) PI(0)	QC(0%) PI(0)												
CUB_BE-MRSA1_R26	QC(0%) PI(0)	QC(0%) PI(0)	QC(0%) PI(0)	QC(0%) PI(0)	QC(98%) PI(99,98)	QC(98%) PI(100)	QC(0%) PI(0)											
CUB_BE-MRSA1_R30	QC(59%) PI(98,23)	QC(59%) PI(98,36)	QC(61%) PI(99,75)	QC(61%) PI(99,77)	QC(0%) PI(0)	QC(0%) PI(0)	QC(99%) PI(99,88)	QC(0%) PI(0)										
CUB_GE-MRSA15_R7	QC(94%) PI(98,24)	QC(89%) PI(98,36)	QC(96%) PI(99,95)	QC(97%) PI(99,96)	QC(0%) PI(0)	QC(0%) PI(0)	QC(52%) PI(94,39)	QC(0%) PI(0)	QC(91%) PI(98,52)									
CUB_GE-MRSA15_R14	QC(93%) PI(97,49)	QC(87%) PI(96,40)	QC(95%) PI(98,63)	QC(94%) PI(98,91)	QC(0%) PI(0)	QC(0%) PI(0)	QC(100%) PI(100)	QC(0%) PI(0)	QC(99%) PI(98,56)	QC(97%) PI(99,17)								
CUB_GE-MRSA15_R23	QC(87%) PI(97,05)	QC(85%) PI(96,70)	QC(89%) PI(99,46)	QC(89%) PI(99,31)	QC(0%) PI(0)	QC(0%) PI(0)	QC(52%) PI(95,77)	QC(0%) PI(0)	QC(99%) PI(98,98)	QC(91%) PI(99,45)	QC(92%) PI(99,39)							
CUB_MRSA-COL_R9	QC(92%) PI(98,44)	QC(90%) PI(98,37)	QC(96%) PI(99,71)	QC(98%) PI(99,77)	QC(0%) PI(0)	QC(0%) PI(0)	QC(47%) PI(92,60)	QC(0%) PI(0)	QC(95%) PI(99,43)	QC(95%) PI(99,71)	QC(96%) PI(98,62)	QC(96%) PI(99,42)						
CUB_MRSA-COL_R20	QC(88%) PI(98,23)	QC(86%) PI(98,36)	QC(91%) PI(99,92)	QC(90%) PI(99,93)	QC(0%) PI(0)	QC(0%) PI(0)	QC(47%) PI(94,62)	QC(0%) PI(0)	QC(98%) PI(99,48)	QC(97%) PI(99,46)	QC(97%) PI(99,16)	QC(97%) PI(99,11)	QC(91%) PI(99,67)					
CUB_MRSA-COL_R23	QC(91%) PI(98,39)	QC(86%) PI(98,46)	QC(91%) PI(99,73)	QC(90%) PI(99,77)	QC(0%) PI(0)	QC(0%) PI(0)	QC(100%) PI(100)	QC(0%) PI(0)	QC(99%) PI(99,22)	QC(97%) PI(99,39)	QC(96%) PI(99,35)	QC(95%) PI(99,00)	QC(91%) PI(99,63)	QC(98%) PI(99,45)				
CUB_GE-MSSA31_R6	QC(0%) PI(0)	QC(0%) PI(0)	QC(0%) PI(0)	QC(0%) PI(0)	QC(98%) PI(99,99)	QC(100%) PI(99.99)	QC(0%) PI(0)	QC(100%) PI(99.99)	QC(0%) PI(0)	QC(0%) PI(0)	QC(0%) PI(0)	QC(0%) PI(0)	QC(0%) PI(0)	QC(0%) PI(0)	QC(0%) PI(0)			
CUB_GE-MSSA31_R16	QC(0%) PI(0)	QC(0%) PI(0)	QC(0%) PI(0)	QC(0%) PI(0)	QC(98%) PI(99,99)	QC(100%) PI(99.99)	QC(0%) PI(0)	QC(100%) PI(99.99)	QC(0%) PI(0)	QC(0%) PI(0)	QC(0%) PI(0)	QC(0%) PI(0)	QC(0%) PI(0)	QC(0%) PI(0)	QC(0%) PI(0)	QC(100%) PI(100%)		
CUB_GE-MSSA31_R27-1	QC(0%) PI(0)	QC(0%) PI(0)	QC(0%) PI(0)	QC(0%) PI(0)	QC(98%) PI(99,98)	QC(100%) PI(99.98)	QC(0%) PI(0)	QC(100%) PI(99.98)	QC(0%) PI(0)	QC(0%) PI(0)	QC(0%) PI(0)	QC(0%) PI(0)	QC(0%) PI(0)	QC(0%) PI(0)	QC(0%) PI(0)	QC(100%) PI(100%)	QC(98%) PI(100%)	
CUB_GE-MSSA31_R27-2	QC(0%) PI(0)	QC(0%) PI(0)	QC(0%) PI(0)	QC(0%) PI(0)	QC(98%) PI(99,98)	QC(100%) PI(99.98)	QC(0%) PI(0)	QC(100%) PI(100)	QC(0%) PI(0)	QC(0%) PI(0)	QC(0%) PI(0)	QC(0%) PI(0)	QC(0%) PI(0)	QC(0%) PI(0)	QC(0%) PI(0)	QC(100%) PI(100%)	QC(100%) PI(100%)	QC(100%) PI(100%)

Table S4. Number of missense mutation identified in the evolved phages compared to the ancestors.

	Ancestral phages																			
	ISP				44AHJD				CUB_A				CUB_B				CUB_M			
Evolved phages	Total	Miss	Kn.Pr	Struc	Total	Miss	Kn.Pr	Struc	Total	Miss	Kn.Pr	Struc	Total	Miss	Kn.Pr	Struc	Total	Miss	Kn.Pr	Struc
CUB_BE-MRSA1_R7	0				3	1	0	0	0				0				0			
CUB_BE-MRSA1_R16	558	83	18	5	2	1	0	0	0				0				0			
CUB_BE-MRSA1_R26	0				4	3	1	0	0				0				0			
CUB_BE-MRSA1_R30	488	83	30	3	0				416	52	26	14	400	65	36	8	378	48	27	11
CUB_GE-MSSA31_R6	0				2	1	0	0	0				0				0			
CUB_GE-MSSA31_R16	0				2	1	0	0	0				0				0			
CUB_GE-MSSA31_R27-1	0				4	2	1	1	0				0				0			
CUB_GE-MSSA31_R27-2	0				4	3	1	0	0				0				0			
CUB_MRSA-COL_R9	547	84	12	6	0				400	118	33	7	413	109	35	1	413	88	19	1
CUB_MRSA-COL_R20	467	100	17	2	0				399	123	36	8	413	118	34	1	413	82	17	7
CUB_MRSA-COL_R20	556	137	23	2	0				421	71	25	14	379	109	71	0	379	82	11	10
CUB_MRSA-COL_R23	565	322	89	3	0				416	77	37	19	377	104	71	0	377	84	13	0
CUB_GE-MRSA15_R7	539	88	4	1	0				405	122	36	9	426	140	51	3	398	93	18	0
CUB_GE-MRSA15_R14	542	95	2	1	0				402	109	36	9	417	106	32	2	411	94	24	16
CUB_GE-MRSA15_R23	544	119	2	1	0				409	66	29	0	379	95	70	0	389	87	53	48

Miss: Missense mutation, Kn.Pr.: known protein, Struc.: structural protein

Table S5. Summary of identified mutations in characterized proteins with known function of evolved phages compared to ancestral phages ISP, 44AHJD and CUB_M.

**Mutations compared to ancestral phage ISP**						
	**Locus**	**Mutation type**	**Reference**	**Alteration**	**Effect on the protein**	**Protein**
**CUB_BE-MRSA1_R26**	Gp72	snp	C	A	missense_variant c.191C>A p.Ala64Glu	Ig-like DCP
	Gp72	snp	G	A	missense_variant c.385G>A p.Val129Ile	Ig-like DCP
	Gp72	complex	TT	CG	missense_variant c.428_429delTTinsCG p.Val143Ala	Ig-like DCP
	Gp72	snp	G	A	missense_variant c.463G>A p.Ala155Thr	Ig-like DCP
	Gp72	snp	T	C	missense_variant c.509T>C p.Val170Ala	Ig-like DCP
**CUB_BE-MRSA1_R30**	Gp9	snp	G	A	missense_variant c.259G>A p.Val87Ile	portal protein
	Gp19	snp	C	A	missense_variant c.1530C>A p.Asp510Glu	major tail sheath protein
	Gp39	snp	A	C	missense_variant c.1745A>C p.Lys582Thr	carbohydrate binding DCP
	Gp72	snp	G	A	missense_variant c.385G>A p.Val129Ile	Ig-like DCP
**CUB_GE-MRSA15_R7**	Gp39	snp	A	C	missense_variant c.1888A>C p.Lys630Gln	carbohydrate binding DCP
**CUB_GE-MRSA15_R14**	Gp39	snp	A	C	missense_variant c.1745A>C p.Lys582Thr	carbohydrate binding DCP
**CUB_MRSA-COL_R9**	Gp9	snp	A	G	missense_variant c.259G>A p.Val87Ile	portal protein
	Gp39	snp	C	A	missense_variant c.1888A>C p.Lys630Gln	carbohydrate binding DCP
**CUB_MRSA-COL_R20**	Gp9	snp	G	A	missense_variant c.259G>A p.Val87Ile	portal protein
	Gp39	snp	A	C	missense_variant c.1745A>C p.Lys582Thr	carbohydrate binding DCP
**CUB_MRSA-COL_R23**	Gp27	snp	A	G	missense_variant c.1737A>G p.Ile579Met	tail-type lysozyme DCP
	Gp27	complex	AGATGCTC AATCC	GGACTCTA AAGAA	missense_variant c.1845_1857delAGATGCTCAATCC	tail-type lysozyme DCP
	Gp39	snp	A	C	missense_variant c.1745A>C p.Lys582Thr	carbohydrate binding DCP
**Mutations compared to ancestral phage CUB-M**						
	**Locus**	**Mutation type**	**Rerefence**	**Alteration**	**Effect on the protein**	**Protein**
**CUB_GE-MRSA15_R23**	Gp87	snp	C	A	missense_variant c.1068G>T p.Lys356Asn	PTMP F
	Gp87	snp	T	C	missense_variant c.731A>G p.Asp244Gly	PTMP F
	Gp87	snp	C	A	missense_variant c.722G>T p.Arg241Ile	PTMP F
	Gp92	snp	A	C	missense_variant c.2519T>G p.Ile840Ser	PTMP F
	Gp92	complex	CG	AT	missense_variant c.2495_2496delCGinsAT p.Thr832Asn	PTMP F
	Gp92	snp	G	A	missense_variant c.2438C>T p.Ala813Val	PTMP F
	Gp92	complex	GC	AA	missense_variant c.2343_2344delGCinsTT p.Glu781Asp	PTMP F
	Gp92	snp	T	G	missense_variant c.2279A>C p.Tyr760Ser	PTMP F
	Gp92	complex	GAGTC	TAGTT	missense_variant c.2109_2113delGACTCinsAACTA p.Leu705Ile	PTMP F
	Gp92	complex	GA	AT	missense_variant c.2051_2052delTCinsAT p.Ile684Asn	PTMP F

Table S5. Continuation

	Locus	Mutation type	Rerefence	Alteration	Effect on the protein	Protein
**Mutations compared to ancestral phage CUB-M**						
**CUB_GE-MRSA15_R23**	Gp92	complex	AATAC	GATAT	missense_variant c.2033_2037delGTATTinsATATC p.Ser678Asn	PTMP F
	Gp92	complex	AGTTAG	GGTTTT	missense_variant c.2011_2016delCTAACTinsAAAACC p.Leu671Lys	PTMP F
	Gp92	snp	T	G	missense_variant c.1996A>C p.Lys666Gln	PTMP F
	Gp92	complex	GTAC	ATAA	missense_variant c.1947_1950delGTACinsTTAT p.Glu649Asp	PTMP F
	Gp92	complex	AGAACCC	GTTACCT	missense_variant c.1929_1935delGGGTTCTinsAGGTAAC p.Ser645Asn	PTMP F
	Gp92	complex	TC	AT	missense_variant c.1923_1924delGAinsAT p.Thr642Ser	PTMP F
	Gp92	mnp	CG	TA	missense_variant c.1788_1789delCGinsTA p.Val597Ile	PTMP F
	Gp92	complex	ATAGTACGT	GCAGTGCGC	missense_variant c.1764_1772delACGTACTATinsGCGCACTGC p.Ile591Ala	PTMP F
	Gp92	snp	C	T	missense_variant c.1745G>A p.Ser582Asn	PTMP F
	Gp92	complex	TCTTA	CTTTG	missense_variant c.1728_1732delTAAGAinsCAAAG p.Arg578Gly	PTMP F
	Gp92	snp	C	T	missense_variant c.1700G>A p.Ser567Asn	PTMP F
	Gp92	complex	CC	TT	missense_variant c.1666_1667delGGinsAA p.Gly556Asn	PTMP F
	Gp92	complex	TT	CC	missense_variant c.1554_1555delAAinsGG p.Lys519Glu	PTMP F
	Gp92	complex	CG	AT	missense_variant c.1529_1530delCGinsAT p.Thr510Asn	PTMP F
	Gp92	snp	C	T	missense_variant c.1486G>A p.Val496Ile	PTMP F
	Gp92	snp	T	C	missense_variant c.1481A>G p.Asn494Ser	PTMP F
	Gp92	complex	TTCAT	CTCAG	missense_variant c.1463_1467delATGAAinsCTGAG p.Asn488Thr	PTMP F
	Gp92	complex	TTGT	CTGC	missense_variant c.1414_1417delACAAinsGCAG p.ThrIle472AlaVal	PTMP F
	Gp92	snp	A	C	missense_variant c.1406T>G p.Ile469Arg	PTMP F
	Gp92	snp	T	C	missense_variant c.1391A>G p.Asn464Ser	PTMP F
	Gp92	complex	AG	CA	missense_variant c.1328_1329delCTinsTG p.Ser443Leu	PTMP F
	Gp92	snp	C	T	missense_variant c.571G>A p.Asp191Asn	PTMP F
**Mutations compared to ancestral phage 44AHJD**						
	**Locus**	**Mutation type**	**Rerefence**	**Alteration**	**Effect on the protein**	**Protein**
**CUB_GE-MSSA31_R27**	ST44AHJD_16	snp	C	T	missense_variant c.1897G>A p.Glu633Lys	minor structural protein

DCP: domain – containing protein, PTMP: putative tail morphogenic protein
